# Greater adherence to the 2019 Canada's Food Guide recommendations on healthy food choices reduces the risk of cardiovascular disease in adults: a prospective analysis of UK Biobank data

**DOI:** 10.1093/ajcn/nqac256

**Published:** 2022-09-16

**Authors:** Didier Brassard, Hasanga D Manikpurage, Sébastien Thériault, Benoît J Arsenault, Benoît Lamarche

**Affiliations:** Center of Nutrition, Health, and Society (NUTRISS), Institute of Nutrition and Functional Foods (INAF), Laval University, Québec, Quebec, Canada; Research Center, University Institute of Cardiology and Pneumology of Quebec, Laval University, Québec, Quebec, Canada; Research Center, University Institute of Cardiology and Pneumology of Quebec, Laval University, Québec, Quebec, Canada; Department of Molecular Biology, Medical Biochemistry, and Pathology, Faculty of Medicine, Laval University, Québec, Quebec, Canada; Research Center, University Institute of Cardiology and Pneumology of Quebec, Laval University, Québec, Quebec, Canada; Department of Medicine, Faculty of Medicine, Laval University, Québec, Quebec, Canada; Center of Nutrition, Health, and Society (NUTRISS), Institute of Nutrition and Functional Foods (INAF), Laval University, Québec, Quebec, Canada

**Keywords:** Healthy Eating Food Index, HEFI-2019, Canada's Food Guide, CFG, cardiovascular disease, CVD, dietary guidelines, causal inference, 24-h dietary recalls

## Abstract

**Background:**

Canada's Food Guide (CFG) was profoundly revised in 2019, but the extent to which adherence to recommendations on healthy food choices reduces the risk of cardiovascular disease (CVD) is unknown.

**Objectives:**

The aim of this study was to examine how greater adherence to the 2019 CFG's recommendations on healthy food choices influences the risk of incident CVD.

**Methods:**

Participants were a sample of adults without history of CVD, diabetes, or cancer from the UK Biobank prospective cohort study. Usual dietary intakes were estimated by modeling data from repeated Web-based 24-h dietary recalls using the National Cancer Institute multivariate method. Adherence to key CFG recommendations on healthy food choices was assessed using the Healthy Eating Food Index (HEFI)-2019, which has a maximum of 80 points. The CVD outcome was a composite of fatal and nonfatal myocardial infarction and ischemic stroke. Cox regression models adjusted via inverse probability weighting were used to estimate CVD risks. Counterfactual models were used to interpret risks of hypothetical changes in the HEFI-2019 score.

**Results:**

A total of 136,698 participants met the eligibility criteria (55% females; mean age: 57.2 y; range: 40–75 y). During the 11-y follow-up, there were 2843 cases of incident CVD. Compared with no change in the HEFI-2019 score, increasing the HEFI-2019 score of all participants to the 90^th^ percentile of the score distribution (58.1 points) hypothetically reduced the risk of CVD by 24% (RR: 0.76; 95% CI: 0.58, 0.94; absolute risk difference: −0.58%).

**Conclusions:**

These results suggest that greater adherence to the 2019 CFG recommendations on healthy food choices reduces the 11-y risk of CVD in middle-aged and older adults.

## Introduction

Diet is an important modifiable risk factor that influences chronic disease–related morbidity and mortality worldwide (1, 2). For example, dietary risks are estimated to account for >14% of cardiovascular disease (CVD)-related mortality in Canada (1). Despite some improvements between 2004 and 2015, the health-related economic burden associated with poor diet quality remains high in Canada (3). National dietary guidelines, including Canada's Food Guide (CFG), aim to address these dietary risks by providing guidance to improve individual dietary habits as well as the food environment and food services.

The CFG was revised in 2019 to promote healthy dietary habits and behaviors aiming to reduce CVD risk (4, 5), based on the most recent evidence on the relation between diet and the risk of chronic diseases (6). Compared with the recommendations in the 2007 version of the CFG, which were stated in terms of sex- and age-specific numbers of food servings to consume every day, CFG-2019 provides more flexible and less specific recommendations. For example, CFG-2019 recommends eating a variety of healthy foods each day (“eat plenty of vegetables and fruits, whole grains and protein foods”) or emphasizes foods and beverages to consume more often (for example, “choose protein foods that come from plants more often,” “make water your drink of choice”), with no specific recommendations based on sex or age (4, 5). However, the extent to which adherence to the 2019 CFG's recommendations does modify the risk of CVD is unknown. Such information is invaluable not only to confirm that adherence to the revised guidelines does indeed reduce the risk of CVD but also to inform future guidelines in Canada, hence further supporting public health action in the realm of food and health.

Thus, our objective was to verify the hypothesis that a higher degree of adherence to CFG's recommendations on healthy food choices reduces the risk of incident CVD. For that purpose, we used publicly available data from the UK Biobank, a large prospective study, as well as the recently developed HEFI-2019, an index that measures the degree of adherence to recommendations on healthy food choices in the 2019 CFG (7, 8). More precisely, we used a causal inference approach based on counterfactual models to emulate a dietary intervention yielding various degrees of adherence to CFG's recommendations on healthy food choices and their impact on incident CVD risk.

## Methods

### Participants and follow-up

Participants were adults from the UK Biobank, which collected data from >500,000 males and females aged between 40 and 69 y recruited in the United Kingdom between 2006 and 2010 (9, 10). At the time of recruitment, participants completed multiple questionnaires and interviews covering sociodemographic data, lifestyle, and history of diseases as well as assessment of physical measures and blood sampling (9). To be included in the present analysis, participants had to be free of CVD, diabetes, or cancer at baseline. Eligible participants also had to have completed at least one 24-h dietary recall reporting ≥100 kcal, to have a urine assay, and to have provided complete data on familial history of disease and physical activity (**Supplemental Table 1**). Baseline was defined as the date when participants completed their first 24-h dietary recall, between the years 2009 and 2012. Follow-up duration was calculated in months using the date of the first incident CVD event, the date of mortality, or end of follow-up (1 February, 2021), whichever came first, minus the baseline date. These analyses were conducted under UK Biobank data application #25205.

### Disease outcome: major CVD events

The primary outcome was a composite of fatal and nonfatal myocardial infarction [International Classification of Diseases (ICD)-10 code I21] and ischemic stroke (ICD-10 code I63). Most CVD events (>75%) were identified based on hospital admission data (**Supplemental Table 2**). The study included no secondary outcomes.

### Diet exposure: adherence to the 2019 CFG on healthy food choices

Detailed dietary intakes were assessed using repeated Web-based 24-h dietary recall intake data obtained with the Oxford WebQ (9, 11, 12). The Oxford WebQ is not based on the Automated Multiple-Pass Method, but rather assesses intakes of ≤206 commonly consumed foods and ≤32 types of beverages (11, 13). All foods and drinks were classified according to the HEFI-2019 food and beverage categories (**Supplemental Figure 1**) (7). Total nutrient intakes were derived from the reported food and beverage intakes and food composition data corresponding to foods available at the time of questionnaire completion. The original food composition database of the Oxford WebQ does not provide data on sodium and free sugar intakes, which contribute to 2 key components of the HEFI-2019. The 24-h sodium intake was estimated based on casual urinary sodium, potassium, and creatinine and the predictive equation of the INTERSALT study (detailed in the **Supplemental Methods**, p. 4) (14). The intake of free sugars was estimated as the difference between total sugars intake and the calculated natural sugars contribution from vegetables, fruits, dairy foods, and legumes (detailed in the Supplemental Methods, p. 4). The HEFI-2019 was used to measure adherence to the 2019 CFG's recommendations on healthy food choices using usual dietary intake data estimated from the 24-h dietary recalls (described in what follows) (7, 8). **Supplemental Table 3** presents a description of components, points, and standards for scoring. The HEFI-2019 has 10 components—5 on foods, 1 on beverages, and 4 on nutrients—each capturing adherence to the key 2019 CFG recommendations on healthy food choices. Points are allocated proportionally between cutoffs that reflect in large part the extent to which intakes of foods within each component are consistent with key CFG recommendations (Supplemental Table 3). The total HEFI-2019 is calculated as the sum of all components’ scores out of a total of 80 points, with higher scores reflecting greater adherence to recommendations. Evaluation metrics of the HEFI-2019 supported construct validity. For example, validation studies based on data from the 2015 Canadian Community Health Survey—Nutrition have shown that the HEFI-2019 score was strongly correlated with the Healthy Eating Index (HEI)-2015 (*r* = 0.79), assessed multiple dimensions of healthy eating, and had acceptable internal consistency (Cronbach's α = 0.66) (8).

### Covariates

Model covariates were selected based on their ability to *1*) mitigate confounding of the relation between the HEFI-2019 score and incident CVD; and *2*) mitigate the impact of an unobserved propensity toward health-seeking behaviors (10). Covariates were sex, age, region, Townsend deprivation index, university degree education, employment status, familial history of CVD, menopausal status (female only), hormone replacement use (female only), smoking habits, physical activity level, alcohol consumption habits, sedentary time, BMI, dietary supplement use, medication use, self-reported CVD risk factors (high cholesterol and/or high blood pressure), and energy intake. The Townsend deprivation index indicates the degree of material deprivation according to census data on unemployment, car ownership, household overcrowding and owner occupation, and the participant's postcode (15). Continuous covariates were transformed a priori as restricted cubic splines (16, 17). A single imputation was performed to account for the remaining proportion of missing data (<2.0%). **Supplemental Table 4** presents complete details regarding covariate sources, modeling, and the proportion with missing data. Covariates were only assessed at baseline.

### Statistical analyses

The causal inference approach based on counterfactuals emulated a dietary intervention (18–20) yielding various degrees of adherence to the 2019 CFG recommendations in this population at baseline and their hypothetical impact on incident CVD. This involved 5 steps to account for measurement error in dietary intakes, mitigate bias due to confounding or censoring, and estimate variance. Further details regarding steps 1–4 are presented in the Supplemental Methods (pp. 11–14).

**FIGURE 1:**
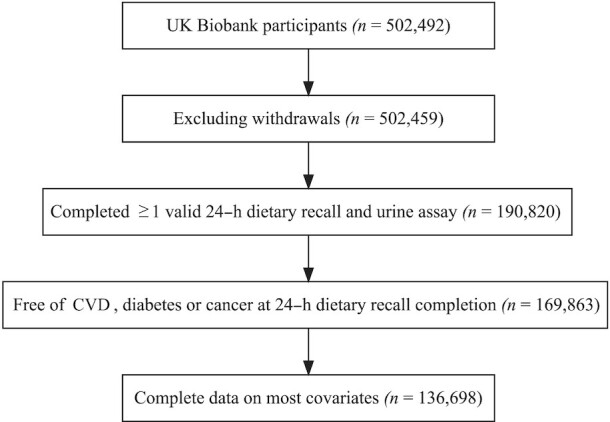
Study flowchart. Valid 24-h dietary recall corresponds to 24-h dietary recall with reported total energy intake ≥100 kcal. Participants with missing data on familial history of CVD or physical activity level were excluded. CVD, cardiovascular disease.

Firstly, the distribution of the HEFI-2019 score was estimated at the population level to identify predetermined HEFI-2019 score percentiles (5^th^, 10^th^, 25^th^, 50^th^, 75^th^, 90^th^, and 95^th^). Dietary intakes measured using 24-h dietary recalls are affected by within-individual random errors (21), which can cause bias (22, 23). To mitigate this issue, we used the National Cancer Institute (NCI)’s multivariate method (24), which estimates the distribution of usual dietary intakes (i.e., the long-term average) when data from repeated 24-h dietary recalls are available in all or only a proportion of the study sample. The large sample size (*n* = 136,698) (**Figure 1**) and the availability of repeated 24-h dietary recalls in most participants (62%) are sufficient to estimate usual dietary intakes with the NCI multivariate method (25).

Secondly, usual dietary intakes were simulated at the participant level with the NCI multivariate method to obtain measurement-error-corrected regression coefficients in diet-outcome models described in what follows. All covariates described earlier were included in this step as well as the predicted 24-h sodium intake based on the urine assay. One thousand simulations of “usual intakes” per participant were generated in the Monte Carlo simulation step of the NCI multivariate method. The total HEFI-2019 score was calculated based on the simulated usual dietary intakes. A restricted cubic spline transformation with 4 knots (percentiles 5, 35, 65, and 95) was applied to the HEFI-2019 score and energy intake to consider a potential nonlinear association with the outcome (26). Simulated usual intakes and HEFI-2019 scores were then averaged across simulations before the next step.

Thirdly, confounding was considered using inverse probability of “treatment” weighting (IPTW) estimated via linear regression (27, 28). The HEFI-2019 score was approximately normally distributed, and a “standard normal” modeling approach was used, i.e., assuming a normal distribution for the total HEFI-2019 score (27). To estimate IPTW, the HEFI-2019 score was regressed on all covariates, except energy intake which was considered as a covariate only in the outcome model in the next step. A probability density function was then estimated based on the total HEFI-2019 score of each individual, predicted values, and variance of the linear regression model. Weights were calculated as the inverse of this probability density function. Informative censoring due to mortality or loss to follow-up was considered with inverse probability of censoring weighting (IPCW) estimated via logistic regression (29). The use of IPCW is a working framework to estimate the (direct) effect of a hypothetical intervention in the presence of competing events (e.g., mortality due to cancer) (29). Weights estimated in both procedures (i.e., IPTW and IPCW) were stabilized to the sample size separately and then combined before the next step. **Supplemental Figure 2** shows a covariate balance plot.

Fourthly, the energy-adjusted relation between the total HEFI-2019 score based on usual intakes and incident CVD was analyzed using Cox proportional hazards regression models. Each participant was weighted using the stabilized inverse probability weights estimated in the previous step to adjust for both confounding and informative censoring. Both the HEFI-2019 score and energy intake were modeled as continuous variables with a restricted cubic spline transformation. The Cox regression model parameters for the HEFI-2019 score and energy intake were then used to generate “adjusted” survival curves (30) at each of the predetermined percentiles of HEFI-2019 score estimated in step 1. The mean energy intake in this sample (2100 kcal) was used for all survival curves, thus ensuring that energy intake remained constant across percentiles of HEFI-2019 score. The impacts of hypothetical changes in the HEFI-2019 score on the 11-y risk of CVD were estimated by calculating relative and absolute differences between the probability of incident CVD, had all participants achieved prespecified percentiles of HEFI-2019 scores, and the probability of incident CVD at the median HEFI-2019 score (i.e., no change; the reference) in this sample. More information on this is provided in the Supplemental Methods (p. 14).

Fifthly, steps 2–4 were repeated independently 250 times to estimate variance via parametric bootstrap. On the one hand, a standard or robust variance estimator may be used to estimate variance in Cox regression models when dietary intake data reflect usual intakes. On the other hand, such an approach would not account for the measurement error correction modeling performed using the NCI method. Thus, we opted for a bootstrap variance estimation to best account for uncertainty at all steps of the NCI multivariate method for measurement error correction (24), uncertainty in the estimation of inverse probability weights (31), as well as variance of the weighted Cox regression model (32).

Finally, sensitivity analyses were conducted to assess the plausibility of reported energy intake (cutoffs shown in **Supplemental Table 5**), to present hazards of CVD across the distribution of HEFI-2019 score using a more traditional approach, to verify proportional hazards of the Cox regression models, and to examine the modification by sex of the relation between the HEFI-2019 score and CVD risk. The E-value was calculated to assess the extent to which an unmeasured confounder could explain the observed effect estimates (33). Analyses were performed in SAS Studio version 3.81 (SAS Institute) and R version 4.1.3 (R Foundation for Statistical Computing).

## Results

### Characteristics of participants

Among the 502,459 participants in the UK Biobank, 136,698 participants met all inclusion criteria (Figure 1). Among them, slightly more than half of participants were females (55.3%) and had a college/university degree or professional qualification (50.4%) (**Supplemental Table 6**). Mean ± SD age was 57.2 ± 8.0 y (range: 40–75 y). Most participants (62%) completed more than one 24-h dietary recall (mean ± SD: 2.2 ± 1.2 recalls). **Table 1** presents characteristics of participants across quarters of total HEFI-2019 score. **Supplemental Tables 7**–**11** present descriptive data on dietary intakes and HEFI-2019 scores.

**TABLE 1:** Baseline characteristics of UK Biobank participants included in this study, by quarters of HEFI-2019 scores^1^

Characteristics	Q1 (min to 38.3) (*n* = 34,174)	Q2 (>38.3 to 45.8) (*n* = 34,175)	Q3 (>45.8 to 52.6) (*n* = 34,175)	Q4 (>52.6 to max) (*n* = 34,174)
Age at dietary assessment, y	55.7 ± 8.2	57.0 ± 8.1	57.7 ± 7.8	58.5 ± 7.6
Sedentary time, h/d	5.2 ± 2.6	4.7 ± 2.3	4.4 ± 2.2	4.1 ± 2.0
BMI, kg/m^2^	27.7 ± 4.6	26.9 ± 4.3	26.3 ± 4.2	25.3 ± 4.0
Females	16.8	22.5	27.4	33.2
Region				
England	24.9	25.0	25.0	25.1
Other	25.9	25.5	24.7	23.8
White/British ethnic background	24.8	25.1	25.1	25.0
Familial history of cardiovascular disease	23.7	24.7	25.3	26.3
College/university degree or professional qualification	21.0	24.5	26.4	28.0
Employment situation				
Working	26.9	25.4	24.5	23.2
Retired	19.8	24.5	26.4	29.3
Other	27.1	23.5	24.5	25.0
Townsend deprivation index				
T1 (min to −3.3)	23.4	25.3	25.7	25.5
T2 (> −3.3 to −1.1)	24.4	25.2	25.3	25.1
T3 (> −1.1 to max)	27.2	24.4	24.0	24.4
Current alcohol consumer	24.7	25.1	25.2	25.0
Current smoker	40.6	24.4	19.7	15.3
BMI ≥ 30 kg/m^2^	34.8	27.1	22.5	15.6
Physical activity level				
Low	33.3	26.7	22.8	17.2
Moderate	24.4	25.4	25.4	24.9
High	22.9	23.9	25.3	27.9
Major dietary habits change in the past 5 y	24.3	25.2	25.0	25.5
Dietary supplement use	20.4	24.0	26.3	29.2
Menopausal status (female only)	13.2	21.0	28.1	37.7
Hormone replacement therapy (female only)	14.2	21.5	28.0	36.3
Self-reported high cholesterol and/or blood pressure	27.2	25.1	24.8	22.9
Plausibility of reported energy intakes				
Under-reporting	35.3	25.2	23.0	16.5
Plausible reporting	24.7	25.4	25.1	24.8
Over-reporting	18.5	23.6	26.0	31.9
Two or more 24-h dietary recalls completed	22.0	25.6	26.4	26.0

1 *n* = 136,698. Values are mean ± SD or row percentages. Row percentages reflect the distribution of a given characteristic across quarters of total HEFI-2019 score. Quartiles of HEFI-2019 scores were estimated based on the average of raw intakes among all 24-h dietary recalls completed. Thus, the quartiles do not reflect usual intakes and misclassification of individuals is expected. Plausible reporting corresponded to a ratio of reported energy intake to predicted energy requirements within 0.74–1.26. See Supplemental Methods p. 9 for details. HEFI-2019, Healthy Eating Food Index 2019; max, maximum value; min, minimum value; Q, quarter.

During the 11-y follow-up, there were 2843 incident cases of fatal and nonfatal CVD (1830 cases of myocardial infarction and 1013 cases of ischemic stroke), for an observed CVD incidence of 2.1%. Among those events, 1971 (69%) occurred in males. A total of 3530 (2.6%) nonoutcome deaths also occurred and 391 (0.3%) participants were lost during the follow-up.

### HEFI-2019 score

**FIGURE 2:**
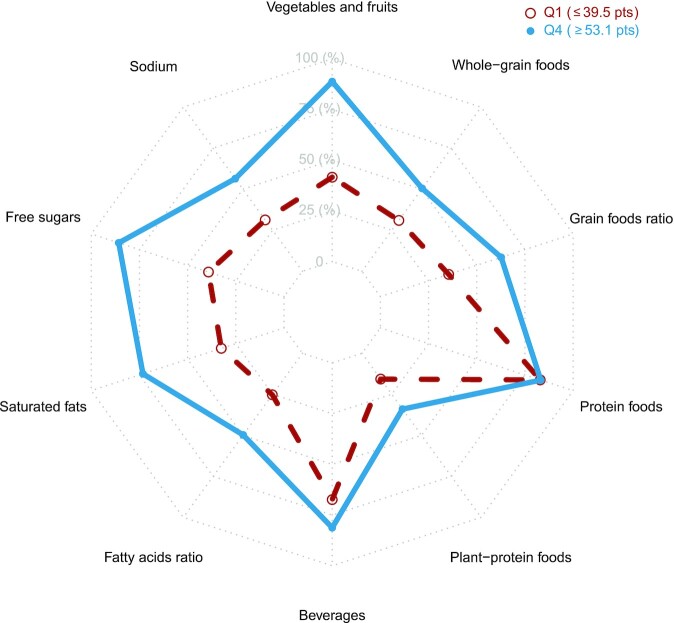
Radar plot depicting mean HEFI-2019 component scores for participants in the lowest and highest quarters of the total HEFI-2019 score distribution in adults from the UK Biobank (*n* = 34,174 each). The plot indicates which component scores contributed to the total HEFI-2019 score among participants with a relatively high total HEFI-2019 score in this sample, compared with participants with a relatively low total HEFI-2019 score. The HEFI-2019 was calculated based on usual dietary intakes collected using 24-h dietary recalls and modeled using the National Cancer Institute's multivariate method (see Methods). Because each component score has a different scale (e.g., Vegetables and fruits is scored on 20 points whereas Protein foods is scored on 5 points), component scores were standardized to percentages, for comparability. HEFI-2019, Healthy Eating Food Index-2019; Q, quarter.

The mean ± SD total HEFI-2019 score on a scale of 80 points was 46.0 ± 9.6 among all participants, 49.0 ± 8.7 in females, and 42.3 ± 9.3 in males. **Figure 2** presents the pattern of HEFI-2019 component scores. Compared with participants in the first quarter of total HEFI-2019 score, participants in the fourth quarter had relatively higher mean HEFI-2019 scores for most of the components, except for Protein foods and Beverages.

### Incident CVD

**FIGURE 3:**
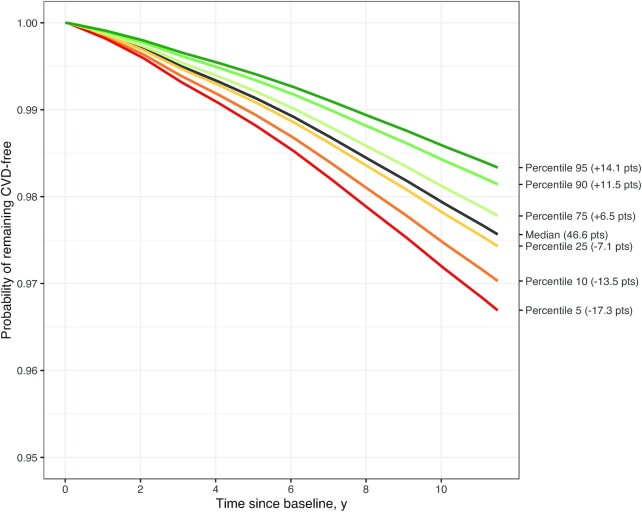
Probability of remaining CVD-free (survival curves) at varying predetermined HEFI-2019 score percentiles in adults from the UK Biobank (*n* = 132,777). The probability of remaining CVD-free at the median HEFI-2019 score is the mean probability and hence is the reference survival curve in a hypothetical scenario where there is no change in the HEFI-2019 score in this population. Other curves reflect the probability of remaining CVD-free under hypothetical scenarios where all participants achieved HEFI-2019 scores corresponding to predetermined percentiles in this population. Estimates of survival probability were based on a fully adjusted Cox regression model using inverse probability weighting for exposure and censoring (see Methods for detail). The HEFI-2019 score was based on usual dietary intakes modeled using the National Cancer Institute multivariate algorithm (see Methods). The total HEFI-2019 score was modeled using a restricted cubic spline with 4 knots. CVD, cardiovascular disease; HEFI-2019, Healthy Eating Food Index-2019.

**Figure 3** presents the survival (CVD-free) probability curves across prespecified percentiles of total HEFI-2019 score. **Table 2** presents the corresponding risk estimates. In this causal inference analysis emulating hypothetical dietary changes, the reference CVD survival (probability of remaining CVD-free) and risks correspond to the scenario where there is no change in the HEFI-2019 score, i.e., at the median (46.6 points) of the score distribution. The other CVD survival curves and corresponding risks reflect hypothetical scenarios where all participants achieved predetermined HEFI-2019 score percentiles at baseline. For example, in a hypothetical intervention where all participants achieved an HEFI-2019 score equivalent to the 90^th^ percentile of the distribution (i.e., 58.1 points, an increase of 11.5 points from the median score), the 11-y RR of CVD would decrease by 24% (RR: 0.76; 95% CI: 0.58, 0.94) (Table 2). Inversely, in an undesirable scenario where all participants ended up with an HEFI-2019 score corresponding to the 10^th^ percentile (i.e., 33.1 points, a decrease of 13.5 points from the median score), the 11-y RR of CVD would increase by 22% (RR: 1.22; 95% CI: 1.03, 1.50) (Table 2).

**TABLE 2:** Estimated risks of CVD in hypothetical scenarios where all eligible participants in the UK Biobank achieved predetermined percentiles of the total HEFI-2019 score at baseline^1^

Total HEFI-2019^2^				Difference in risk estimates (95% CI)	
Percentile	Score (/80)	Hypothetical change	11-y CVD risk	Absolute, % point	Relative
95	60.7	+14.1 pts	1.7%	−0.77 (−1.43, −0.11)	0.68 (0.43, 0.93)
90	58.1	+11.5 pts	1.9%	−0.58 (−1.05, −0.10)	0.76 (0.58, 0.94)
75	53.1	+6.5 pts	2.2%	−0.21 (−0.44, 0.01)	0.91 (0.82, 1.00)
50	46.6	0 pts (reference)	2.4%	0 (reference)	1 (reference)
25	39.5	−7.1 pts	2.6%	0.14 (−0.27, 0.55)	1.06 (0.91, 1.26)
10	33.1	−13.5 pts	3.0%	0.54 (0.04, 1.04)	1.22 (1.03, 1.50)
5	29.3	−17.3 pts	3.3%	0.87 (0.18, 1.57)	1.36 (1.10, 1.78)

1 *n* = 132,777. Differences in risk estimates reflect risks, had all participants in the sample achieved a prespecified HEFI-2019 score percentile, compared with the risk at the median HEFI-2019 score among all participants, the reference scenario where there is no change in HEFI-2019 (i.e., “0 pts”). Estimates were based on a fully adjusted Cox regression model using inverse probability weighting for dietary exposure and censoring. The inverse probability weighting model covariates were sex, age, region, Townsend deprivation index, university degree, employment, familial history of CVD, menopausal status (female only), hormone replacement use (female only), smoking habits, physical activity level, alcohol consumption habits, sedentary time, BMI, dietary supplement use, medication use, and self-reported risk factor (high cholesterol and/or high blood pressure). Energy intake was included as a covariate in the Cox regression model. The 95% CIs were estimated using 250 bootstrap samples. CVD, cardiovascular disease; HEFI-2019, Healthy Eating Food Index-2019.

2 The HEFI-2019 score was based on usual dietary intakes modeled using the National Cancer Institute multivariate algorithm (see Methods). The HEFI-2019 score was modeled using a restricted cubic spline with 4 knots.

### Sensitivity analyses

**FIGURE 4:**
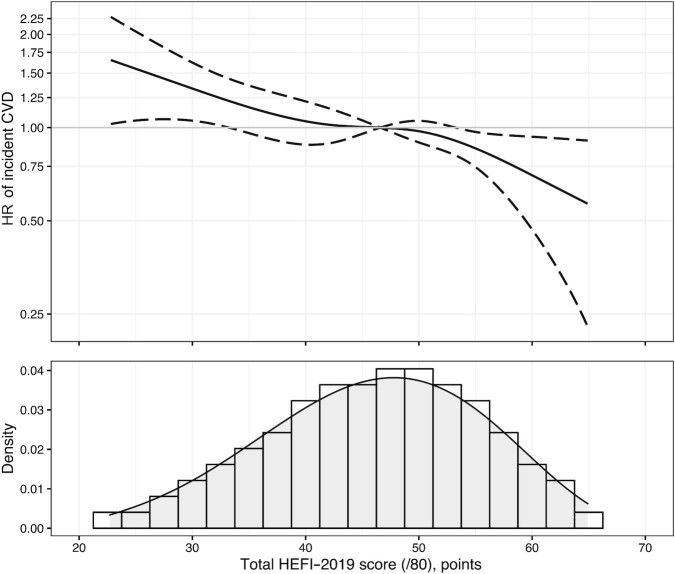
Hazard ratio (HR) curve of CVD (top panel) along the total HEFI-2019 score distribution (lower panel) in adults from the UK Biobank (*n* = 132,777). The reference solid line (HR = 1.0 in the top panel) corresponds to the median HEFI-2019 total score in this population. The HR curve was based on a fully adjusted Cox regression model using inverse probability weighting for exposure and censoring. The HEFI-2019 score was based on usual dietary intakes modeled using the National Cancer Institute multivariate algorithm (see Methods). The total HEFI-2019 score was modeled using a restricted cubic spline with 4 knots. The plot was restricted to percentiles 1–99 of the total HEFI-2019 score to avoid extrapolation of hazards beyond observed scores (i.e., 22.7–64.9 points). The 95% CIs were estimated using 250 bootstrap samples. CVD, cardiovascular disease; HEFI-2019, Healthy Eating Food Index-2019; /80, maximum score possible for the Healthy Eating Food Index-2019.

The total HEFI-2019 score was inversely associated with hazards of CVD across its distribution (**Figure 4**), consistent with the differences in risk estimates presented earlier in the causal inference analysis. CVD hazards at varying lengths of follow-up were generally consistent over time (**Supplemental Table 12**). CVD survival curves and risks based on a fully parametric modeling of the time to CVD were similar to the survival curves based on the Cox regression models (**Supplemental Figure 3, Supplemental Table 13**). There was no evidence that the RR of CVD according to hypothetical changes in HEFI-2019 score was modified by sex (**Table 3**, **Supplemental Figure 4**).

**TABLE 3:** Estimated risks of CVD in hypothetical scenarios where all eligible participants in the UK Biobank achieved predetermined percentiles of the total HEFI-2019 score at baseline, by sex^1^

Total HEFI-2019^2^				Difference in risk estimates (95% CI)		
Percentile^3^	Score (/80)	Hypothetical change	11-y CVD risk	Absolute, % point	Relative	*P*-interaction^4^
Females (*n* = 73,811)						
90	58.1	+11.5 pts	1.3%	−0.17 (−0.50, 0.17)	0.89 (0.68, 1.09)	—
75	53.1	+6.5 pts	1.3%	−0.12 (−0.36, 0.13)	0.92 (0.76, 1.08)	—
50	46.6	0 pts (reference)	1.5%	0 (reference)	1 (reference)	—
25	39.5	−7.1 pts	1.8%	0.29 (−0.09, 0.67)	1.20 (0.98, 1.53)	—
10	33.1	−13.5 pts	2.2%	0.73 (−0.09, 1.55)	1.50 (1.03, 2.73)	—
Males (*n* = 58,966)						
90	58.1	+11.5 pts	2.6%	−0.86 (−1.70, −0.02)	0.75 (0.53, 0.97)	*P* = 0.27
75	53.1	+6.5 pts	3.1%	−0.33 (−0.73, 0.07)	0.90 (0.79, 1.02)	*P* = 0.87
50	46.6	0 pts (reference)	3.5%	0 (reference)	1 (reference)	—
25	39.5	−7.1 pts	3.6%	0.17 (−0.50, 0.84)	1.05 (0.88, 1.30)	*P* = 0.32
10	33.1	−13.5 pts	4.1%	0.65 (0.02, 1.27)	1.19 (1.01, 1.43)	*P* = 0.17

1 *n* = 132,777. Difference in risk estimates reflect risks, had all participants in the sample achieved a prespecified HEFI-2019 score percentile, compared with the risk at the median HEFI-2019 score among all participants, the reference scenario where there is no change in HEFI-2019 (i.e., “0 pts”). Estimates were based on fully adjusted pooled logistic regression models using inverse probability weighting for dietary exposure and censoring and stratified by sex. In the pooled logistic regression model, the time to CVD was modeled using a restricted cubic spline with 5 knots and an interaction term between the total HEFI-2019 score and time to CVD was included as well as total energy intake. The inverse probability weighting models were also stratified by sex and covariates were age, region, Townsend deprivation index, university degree, employment, familial history of CVD, menopausal status (female only), hormone replacement use (female only), smoking habits, physical activity level, alcohol consumption habits, sedentary time, BMI, dietary supplement use, medication use, and self-reported risk factor (high cholesterol and/or high blood pressure). The 95% CIs were estimated using 250 bootstrap samples. CVD, cardiovascular disease; HEFI-2019, Healthy Eating Food Index-2019.

2 The HEFI-2019 score was based on usual dietary intakes modeled using the National Cancer Institute multivariate algorithm (see Methods). The HEFI-2019 score was modeled using a restricted cubic spline with 4 knots.

3 Percentile values are not sex-specific, i.e., they reflect the combined distribution of total HEFI-2019 scores of males and females.

4 *P* values for interaction (*t* test) reflect the compatibility of the RR in males compared with the RR in females for a given percentile, with the (null) hypothesis that both RRs are equivalent under this model.

## Discussion

The objective of this study was to verify the hypothesis that a greater degree of adherence to the 2019 CFG's recommendations on healthy food choices, measured with the HEFI-2019, reduces the risk of incident CVD. A causal inference analysis emulating changes in HEFI-2019 scores under hypothetical intervention scenarios among >130,000 adult participants of the UK Biobank supports this hypothesis. These results provide evidence supporting the legitimacy and usefulness of the 2019 CFG recommendations on healthy food choices to reduce CVD risk at the population level.

Key CFG recommendations on healthy food choices aim at increasing intake of vegetables, fruits, whole grains, plant-based protein foods, water and unsweetened beverages, and unsaturated fats, while decreasing the relative proportion of refined grains, sugary drinks, and processed meats, as well as saturated fats, free sugars, and sodium intakes (4, 5, 7). These recommendations are common to many other dietary guidelines around the world (34) and are also key features of well-established healthy patterns such as the Mediterranean diet (35, 36). Few randomized dietary intervention trials have been conducted to date wherein causality can be documented with more certainty. Among them, the PREDIMED (Prevención con Dieta Mediterránea) randomized controlled trial has shown that participants randomly assigned to a Mediterranean diet supplemented with either extra-virgin olive oil or mixed nuts had a 30% lower hazard of CVD over a 4.8-y follow-up than participants randomly assigned to the control diet (37). More recently, the CORDIOPREV (Coronary Diet Intervention with Olive Oil and Cardiovascular Prevention) randomized controlled trial confirmed that the Mediterranean diet was superior to the low-fat diet in preventing major cardiovascular events in secondary prevention patients (38). Using observational data, others have examined the relation between adherence to the Dietary Guidelines for Americans using HEIs and incident CVD. Participants with Alternative HEI-2010 and HEI-2015 scores above the fifth quintile, reflecting a greater degree of adherence to the dietary guidelines, had a 24% and 16% lower hazard of incident CVD, respectively, than participants whose scores were below the first quintile (39, 40). Another analysis based on multiple cohorts has found consistent reduction in hazards of CVD mortality in participants with higher diet quality (above the fifth quintile), as measured with the Alternative HEI-2010, the HEI-2010 score, or the alternative Mediterranean diet score (41). Finally, a systematic review and meta-analysis pooling different diet quality scores found a 20% reduced risk (95% CI: 0.78, 0.82) of CVD incidence or mortality with higher diet quality scores (42). Dietary guidelines are crafted and updated regularly to consider the most recent evidence associating a variety of dietary patterns, foods, and nutrients to health, with a focus generally on the risk of chronic diseases such as CVD, cancer, and type 2 diabetes. Considering the similarities among various sets of dietary guidelines and the rigorous process implemented for their update, it is unsurprising that higher adherence to such guidelines, including those found in the 2019 CFG, has been systematically associated with a reduced risk of CVD.

A key assumption of causal effect estimation using observational data is that there is no unmeasured confounding (18, 43). However, as shown in the Supplemental Methods (p. 27), residual (or unmeasured) confounding would have to be relatively important to completely “nullify” the lower RR of CVD at higher HEFI-2019 scores. Moreover, follow-up data collection among all participants was not implemented in the UK Biobank and changes in dietary intakes and potential time-varying confounders such as incident risk factors and change in medication could not be considered. Data from this study must therefore be interpreted as an observational analog of an intention-to-treat effect in which nonadherence to the hypothetical intervention is expected but could not be accounted for. Future studies with repeated data collection including diet will help provide more accurate estimates of how adherence to CFG recommendations influences the risk of CVD by considering nonadherence, i.e., estimating a per-protocol effect (18, 19, 44).

The differences in dietary habits between the United Kingdom and Canada and related considerations need to be addressed in the larger scope of this work on the HEFI-2019, a Canadian-focused tool. Firstly, the associations between specific dietary risk factors such as low intake of vegetables and fruits, low intake of fibers, or high intake of meat, saturated fats, or free sugars and cardiovascular outcomes have been observed in diverse populations around the world, including in the United Kingdom (34, 45–48). The pathophysiology of diet-related CVD is also unlikely to differ markedly between Canada and the United Kingdom. Yet, it is possible that the magnitude of the associations between the dietary risk factors captured by the various HEFI-2019 components and CVD risk varies across different populations. Nevertheless, we are confident that results from this analysis substantiate the relevance of the recommendations on healthy food choices as stated in the CFG-2019 to prevent CVD. Secondly, the extent to which this analysis based on a sample of UK adults under- or overestimates the actual reduction in CVD risk associated with higher HEFI-2019 scores among Canadian adults also remains uncertain. Thirdly, the external validity of the CVD risk estimates also needs to be interpreted while recognizing that adults from the UK Biobank are generally more health-conscious than other adults in the United Kingdom (10). Accordingly, the crude absolute 11-y risk of CVD was very low in this sample (2.1%). This suggests that the absolute CVD risk reductions associated with higher HEFI-2019 scores in this eligible sample of the UK Biobank may have been underestimated compared with the risk reductions that would have been observed in a less health-conscious population.

One of the major strengths of this work relates to the use of a counterfactual framework that addresses causality by deriving realistic HEFI-2019 score contrasts emulating a dietary intervention. A more conventional approach to assessing the CVD hazards associated with variations in the HEFI-2019 score in the study population yielded results that were highly consistent, further supporting the validity of the results from the causal inference analyses. Accounting for random errors in the assessment of the dietary intakes measured with 24-h dietary recalls with the NCI multivariate method is also a key strength of this work. Limitations of this work include the challenge in measuring dietary habits as the exposure variable because 24-h dietary recalls are known to be affected by systematic errors, although to a lesser extent than other common instruments such as FFQs (49–51). Of note, the extent of potential under-reporting of total energy intakes in the present study (i.e., 14.6%) is not greater than what has been reported in other contemporary nutrition surveys (52, 53). Finally, unmeasured confounding is a limitation as in most observational studies.

In conclusion, emulating a large dietary intervention within the UK Biobank provided strong evidence that a greater degree of adherence to the CFG's recommendations on healthy food choices, as measured with the HEFI-2019, reduces the 11-y risk of major CVD in middle-aged and older adults. To best inform future dietary guidelines in Canada, studies and analyses in Canadian cohorts are needed: firstly, to confirm these results and, secondly, to better ascertain the magnitude of the CVD risk reduction associated with better diet quality in Canada.

## Supplementary Material

nqac256_Supplemental_File

## Data Availability

The UK Biobank is an open access resource. Data described in the article and code book are available upon request by registering and applying at http://www.ukbiobank.ac.uk/register-apply. The analytic code will be made publicly and freely available without restriction at https://github.com/didierbrassard/hefi2019_cvd.

## Notes

The authors reported no funding received for this study.

Author disclosures: DB was a casual employee of Health Canada (2019–2020), held a doctoral training award from the Fonds de recherche du Québec—Santé (FRQS) (2019–2021), and holds Canadian Institutes of Health Research (CIHR) Fellowship award MFE-181852. BJA is a consultant for Novartis and Silence Therapeutics and has received research contracts from Pfizer, Ionis Pharmaceuticals, and Silence Therapeutics. BL has received funding from the CIHR (ongoing), the FRQS (ongoing), Fonds de recherche du Québec—Nature et technologies (NT) (ongoing), the Ministère de la santé et des services sociaux (MSSS) du Québec (ongoing), Health Canada (completed in 2021), and Atrium Innovations (completed in 2019). BL is an Advisory Board member of the Canadian Nutrition Society. All other authors report no conflicts of interest.

Supplemental Methods, Supplemental Figures 1–4, and Supplemental Tables 1–13 are available from the “Supplementary data” link in the online posting of the article and from the same link in the online table of contents at https://academic.oup.com/ajcn/.

Address correspondence to BL (e-mail: benoit.lamarche@fsaa.ulaval.ca).

Abbreviations used: CFG, Canada's Food Guide; CVD, cardiovascular disease; HEFI-2019, Healthy Eating Food Index-2019; HEI, Healthy Eating Index; ICD, International Classification of Diseases; IPCW, inverse probability of censoring weighting; IPTW, inverse probability of “treatment” weighting; NCI, National Cancer Institute.

## References

[1] Alam S , Lang JJ, Drucker AM, Gotay C, Kozloff N, Mate K et al. Assessment of the burden of diseases and injuries attributable to risk factors in Canada from 1990 to 2016: an analysis of the Global Burden of Disease Study. CMAJ Open. 2019;7(1):E140–E8. 10.9778/cmajo.20180137 PMC6397034 30819694

[2] GBD Risk Factors Collaborators . Global burden of 87 risk factors in 204 countries and territories, 1990–2019: a systematic analysis for the Global Burden of Disease Study 2019. Lancet. 2020;396(10258):1223–49. 33069327 10.1016/S0140-6736(20)30752-2 PMC7566194

[3] Nshimyumukiza L , Lieffers JRL, Ekwaru JP, Ohinmaa A, Veugelers PJ. Temporal changes in diet quality and the associated economic burden in Canada. PLoS One. 2018;13(11):e0206877. 30408076 10.1371/journal.pone.0206877 PMC6224068

[4] Health Canada . Canada's Food Guide. Ottawa, Canada: Health Canada; 2019.

[5] Health Canada . Canada's Dietary Guidelines - for health professionals and policy makers. Ottawa, Canada: Health Canada; 2019.

[6] Health Canada . Food, nutrients and health: interim evidence update 2018 for health professionals and policy makers. Ottawa, Canada: Health Canada; 2019.

[7] Brassard D , Elvidge Munene LA, St-Pierre S, Guenther PM, Kirkpatrick SI, Slater J et al. Development of the Healthy Eating Food Index (HEFI)-2019 measuring adherence to Canada's Food Guide 2019 recommendations on healthy food choices. Appl Physiol Nutr Metab. 2022;47(5):595–610. 35030038 10.1139/apnm-2021-0415

[8] Brassard D , Elvidge Munene L-A, St-Pierre S, Gonzalez A, Guenther PM, Jessri M et al. Evaluation of the Healthy Eating Food Index (HEFI)-2019 measuring adherence to Canada's Food Guide 2019 recommendations on healthy food choices. Appl Physiol Nutr Metab. 2022;47(5):582–94. 35030069 10.1139/apnm-2021-0416

[9] Sudlow C , Gallacher J, Allen N, Beral V, Burton P, Danesh J et al. UK Biobank: an open access resource for identifying the causes of a wide range of complex diseases of middle and old age. PLoS Med. 2015;12(3):e1001779. 25826379 10.1371/journal.pmed.1001779 PMC4380465

[10] Fry A , Littlejohns TJ, Sudlow C, Doherty N, Adamska L, Sprosen T et al. Comparison of sociodemographic and health-related characteristics of UK Biobank participants with those of the general population. Am J Epidemiol. 2017;186(9):1026–34. 28641372 10.1093/aje/kwx246 PMC5860371

[11] Liu B , Young H, Crowe FL, Benson VS, Spencer EA, Key TJ et al. Development and evaluation of the Oxford WebQ, a low-cost, web-based method for assessment of previous 24 h dietary intakes in large-scale prospective studies. Public Health Nutr. 2011;14(11):1998–2005. 21729481 10.1017/S1368980011000942

[12] Greenwood DC , Hardie LJ, Frost GS, Alwan NA, Bradbury KE, Carter M et al. Validation of the Oxford WebQ online 24-hour dietary questionnaire using biomarkers. Am J Epidemiol. 2019;188(10):1858–67. 31318012 10.1093/aje/kwz165 PMC7254925

[13] Perez-Cornago A , Pollard Z, Young H, van Uden M, Andrews C, Piernas C et al. Description of the updated nutrition calculation of the Oxford WebQ questionnaire and comparison with the previous version among 207,144 participants in UK Biobank. Eur J Nutr. 2021;60(7):4019–30. 33956230 10.1007/s00394-021-02558-4 PMC8437868

[14] Brown IJ , Dyer AR, Chan Q, Cogswell ME, Ueshima H, Stamler J et al. Estimating 24-hour urinary sodium excretion from casual urinary sodium concentrations in Western populations: the INTERSALT study. Am J Epidemiol. 2013;177(11):1180–92. 23673246 10.1093/aje/kwt066 PMC3664342

[15] Townsend P , Phillimore P, Beattie A. Health and deprivation: inequality and the north. London, United Kingdom: Croom Helm; 1987.

[16] Kyle RP , Moodie EEM, Klein MB, Abrahamowicz M. Evaluating flexible modeling of continuous covariates in inverse-weighted estimators. Am J Epidemiol. 2019;188(6):1181–91. 30649165 10.1093/aje/kwz004 PMC6545287

[17] Harrell FE Jr . General aspects of fitting regression models. In: Regression modeling strategies: with applications to linear models, logistic and ordinal regression, and survival analysis. Cham, Switzerland: Springer International Publishing; 2015. p. 13–44.

[18] Chiu Y-H , Chavarro JE, Dickerman BA, Manson JE, Mukamal KJ, Rexrode KM et al. Estimating the effect of nutritional interventions using observational data: the American Heart Association's 2020 Dietary Goals and mortality. Am J Clin Nutr. 2021;114(2):690–703. 34041538 10.1093/ajcn/nqab100 PMC8326054

[19] Kutcher SA , Brophy JM, Banack HR, Kaufman JS, Samuel M. Emulating a randomised controlled trial with observational data: an introduction to the target trial framework. Can J Cardiol. 2021;37(9):1365–77. 34090982 10.1016/j.cjca.2021.05.012

[20] Tobias DK , Lajous M. What would the trial be? Emulating randomized dietary intervention trials to estimate causal effects with observational data. Am J Clin Nutr. 2021;114(2):416–17. 34041528 10.1093/ajcn/nqab169

[21] Thompson FE , Kirkpatrick SI, Subar AF, Reedy J, Schap TE, Wilson MM et al. The National Cancer Institute's Dietary Assessment Primer: a resource for diet research. J Acad Nutr Diet. 2015;115(12):1986–95. 26422452 10.1016/j.jand.2015.08.016 PMC4663113

[22] Kipnis V , Midthune D, Buckman DW, Dodd KW, Guenther PM, Krebs-Smith SM et al. Modeling data with excess zeros and measurement error: application to evaluating relationships between episodically consumed foods and health outcomes. Biometrics. 2009;65(4):1003–10. 19302405 10.1111/j.1541-0420.2009.01223.x PMC2881223

[23] Brakenhoff TB , van Smeden M, Visseren FLJ, Groenwold RHH. Random measurement error: why worry? An example of cardiovascular risk factors. PLoS One. 2018;13(2):e0192298. 29425217 10.1371/journal.pone.0192298 PMC5806872

[24] Zhang S , Midthune D, Guenther PM, Krebs-Smith SM, Kipnis V, Dodd KW et al. A new multivariate measurement error model with zero-inflated dietary data, and its application to dietary assessment. Ann Appl Stat. 2011;5(2B):1456–87. 21804910 10.1214/10-AOAS446 PMC3145332

[25] Kirkpatrick SI , Guenther PM, Subar AF, Krebs-Smith SM, Herrick KA, Freedman LS et al. Using short-term dietary intake data to address research questions related to usual dietary intake among populations and subpopulations: assumptions, statistical techniques, and considerations. J Acad Nutr Diet. 2022;122(7):1246–62. 35283362 10.1016/j.jand.2022.03.010

[26] Desquilbet L , Mariotti F. Dose-response analyses using restricted cubic spline functions in public health research. Stat Med. 2010;29(9):1037–57. 20087875 10.1002/sim.3841

[27] Naimi AI , Moodie EE, Auger N, Kaufman JS. Constructing inverse probability weights for continuous exposures: a comparison of methods. Epidemiology. 2014;25(2):292–9. 24487212 10.1097/EDE.0000000000000053

[28] Cole SR , Hernan MA. Constructing inverse probability weights for marginal structural models. Am J Epidemiol. 2008;168(6):656–64. 18682488 10.1093/aje/kwn164 PMC2732954

[29] Young JG , Stensrud MJ, Tchetgen Tchetgen EJ, Hernan MA. A causal framework for classical statistical estimands in failure-time settings with competing events. Stat Med. 2020;39(8):1199–236. 31985089 10.1002/sim.8471 PMC7811594

[30] Cole SR , Hernan MA. Adjusted survival curves with inverse probability weights. Comput Methods Programs Biomed. 2004;75(1):45–9. 15158046 10.1016/j.cmpb.2003.10.004

[31] Austin PC . Variance estimation when using inverse probability of treatment weighting (IPTW) with survival analysis. Stat Med. 2016;35(30):5642–55. 27549016 10.1002/sim.7084 PMC5157758

[32] Stensrud MJ , Hernan MA. Why test for proportional hazards?. JAMA. 2020;323(14):1401–2. 32167523 10.1001/jama.2020.1267 PMC11983487

[33] VanderWeele TJ , Ding P. Sensitivity analysis in observational research: introducing the E-value. Ann Intern Med. 2017;167(4):268–74. 28693043 10.7326/M16-2607

[34] Kebbe M , Gao M, Perez-Cornago A, Jebb SA, Piernas C. Adherence to international dietary recommendations in association with all-cause mortality and fatal and non-fatal cardiovascular disease risk: a prospective analysis of UK Biobank participants. BMC Med. 2021;19(1):134. 34158032 10.1186/s12916-021-02011-7 PMC8220774

[35] Millen BE , Abrams S, Adams-Campbell L, Anderson CA, Brenna JT, Campbell WW et al. The 2015 Dietary Guidelines Advisory Committee scientific report: development and major conclusions. Adv Nutr. 2016;7(3):438–44. 27184271 10.3945/an.116.012120 PMC4863277

[36] Lloyd-Jones DM , Hong Y, Labarthe D, Mozaffarian D, Appel LJ, Van Horn L et al. Defining and setting national goals for cardiovascular health promotion and disease reduction: the American Heart Association's strategic Impact Goal through 2020 and beyond. Circulation. 2010;121(4):586–613. 20089546 10.1161/CIRCULATIONAHA.109.192703

[37] Estruch R , Ros E, Salas-Salvadó J, Covas M-I, Corella D, Arós F et al. Primary prevention of cardiovascular disease with a Mediterranean diet supplemented with extra-virgin olive oil or nuts. N Engl J Med. 2018;378(25):e34. 29897866 10.1056/NEJMoa1800389

[38] Delgado-Lista J , Alcala-Diaz JF, Torres-Peña JD, Quintana-Navarro GM, Fuentes F, Garcia-Rios A et al. Long-term secondary prevention of cardiovascular disease with a Mediterranean diet and a low-fat diet (CORDIOPREV): a randomised controlled trial. Lancet. 2022;399(10338):1876–85. 35525255 10.1016/S0140-6736(22)00122-2

[39] Hu EA , Steffen LM, Coresh J, Appel LJ, Rebholz CM. Adherence to the Healthy Eating Index–2015 and other dietary patterns may reduce risk of cardiovascular disease, cardiovascular mortality, and all-cause mortality. J Nutr. 2020;150(2):312–21. 31529069 10.1093/jn/nxz218 PMC7373820

[40] Chiuve SE , Fung TT, Rimm EB, Hu FB, McCullough ML, Wang M et al. Alternative dietary indices both strongly predict risk of chronic disease. J Nutr. 2012;142(6):1009–18. 22513989 10.3945/jn.111.157222 PMC3738221

[41] Liese AD , Krebs-Smith SM, Subar AF, George SM, Harmon BE, Neuhouser ML et al. The Dietary Patterns Methods Project: synthesis of findings across cohorts and relevance to dietary guidance. J Nutr. 2015;145(3):393–402. 25733454 10.3945/jn.114.205336 PMC4336525

[42] Morze J , Danielewicz A, Hoffmann G, Schwingshackl L. Diet quality as assessed by the Healthy Eating Index, Alternate Healthy Eating Index, Dietary Approaches to Stop Hypertension score, and health outcomes: a second update of a systematic review and meta-analysis of cohort studies. J Acad Nutr Diet. 2020;120(12):1998–2031.e15. 33067162 10.1016/j.jand.2020.08.076

[43] Hernán MA , Robins JM. Causal inference: what if. Boca Raton, FL: Chapman & Hall/CRC; 2020.

[44] Hernan MA , Robins JM. Using big data to emulate a target trial when a randomized trial is not available. Am J Epidemiol. 2016;183(8):758–64. 26994063 10.1093/aje/kwv254 PMC4832051

[45] Papier K , Fensom GK, Knuppel A, Appleby PN, Tong TYN, Schmidt JA et al. Meat consumption and risk of 25 common conditions: outcome-wide analyses in 475,000 men and women in the UK Biobank study. BMC Med. 2021;19(1):53. 33648505 10.1186/s12916-021-01922-9 PMC7923515

[46] Wang M , Ma H, Song Q, Zhou T, Hu Y, Heianza Y et al. Red meat consumption and all-cause and cardiovascular mortality: results from the UK Biobank study. Eur J Nutr. 2022;61(5):2543–53. 35220441 10.1007/s00394-022-02807-0

[47] Kelly RK , Watling CZ, Tong TYN, Piernas C, Carter JL, Papier K et al. Associations between macronutrients from different dietary sources and serum lipids in 24 639 UK Biobank study participants. Arterioscler Thromb Vasc Biol. 2021;41(7):2190–200. 34039019 10.1161/ATVBAHA.120.315628 PMC8216602

[48] Brayner B , Keske MA, Kaur G, Islam SMS, Perez-Cornago A, Piernas C et al. Longitudinal associations between fat-derived dietary patterns and early markers of cardiovascular disease risk in the UK Biobank study. J Am Heart Assoc. 2022;11(11):e024069. 35621194 10.1161/JAHA.121.024069 PMC9238710

[49] Freedman LS , Commins JM, Moler JE, Willett W, Tinker LF, Subar AF et al. Pooled results from 5 validation studies of dietary self-report instruments using recovery biomarkers for potassium and sodium intake. Am J Epidemiol. 2015;181(7):473–87. 25787264 10.1093/aje/kwu325 PMC4371766

[50] Freedman LS , Commins JM, Moler JE, Arab L, Baer DJ, Kipnis V et al. Pooled results from 5 validation studies of dietary self-report instruments using recovery biomarkers for energy and protein intake. Am J Epidemiol. 2014;180(2):172–88. 24918187 10.1093/aje/kwu116 PMC4082341

[51] Park Y , Dodd KW, Kipnis V, Thompson FE, Potischman N, Schoeller DA et al. Comparison of self-reported dietary intakes from the Automated Self-Administered 24-h recall, 4-d food records, and food-frequency questionnaires against recovery biomarkers. Am J Clin Nutr. 2018;107(1):80–93. 29381789 10.1093/ajcn/nqx002 PMC5972568

[52] Murakami K , Livingstone MB. Prevalence and characteristics of misreporting of energy intake in US adults: NHANES 2003–2012. Br J Nutr. 2015;114(8):1294–303. 26299892 10.1017/S0007114515002706

[53] Garriguet D . Accounting for misreporting when comparing energy intake across time in Canada. Health Rep. 2018;29(5):3–12. 29852052

