# Supplementary material for: Greater adherence to the 2019 Canada's Food Guide recommendations on healthy food choices reduces the risk of cardiovascular disease in adults: a prospective analysis of UK Biobank data
Source: Am J Clin Nutr. 2022 Sep 16;116(6):1748–58. doi: 10.1093/ajcn/nqac256 (PMC9761735; doi:10.1093/ajcn/nqac256)
Supplement: nqac256_Supplemental_File [file nqac256_supplemental_file.docx]

**Supplementary Material**

**Title**: Greater adherence to the 2019 Canada’s Food Guide recommendations on healthy food choices reduces the risk of cardiovascular disease in adults: a prospective analysis of UK Biobank data

**Authors** : Brassard, Didier; Manikpurage, Hasanga D.; Thériault, Sébastien; Arsenault, Benoît J.; Lamarche, Benoît

Table of Contents

[Supplementary methods 1](#_Toc109922037)

[Estimand and hypothetical changes 1](#_Toc109922038)

[Detailed description of exclusion criteria 2](#_Toc109922039)

[Source of CVD outcome data 3](#_Toc109922040)

[The Healthy Eating Food Index (HEFI)-2019 4](#_Toc109922041)

[Classification of foods in the Oxford WebQ 6](#_Toc109922042)

[Covariates 7](#_Toc109922043)

[Plausibility of self-reported energy intakes 9](#_Toc109922044)

[Statistical analyses 11](#_Toc109922045)

[Steps 1 and 2: Modelling of dietary constituents. 11](#_Toc109922046)

[Step 3: Adjustments using inverse probability weighting (IPW) 12](#_Toc109922047)

[Step 4: outcome model for incident CVD 14](#_Toc109922048)

[Supplementary results 15](#_Toc109922049)

[Characteristics of participants 15](#_Toc109922050)

[Dietary intakes 18](#_Toc109922051)

[Relationship between the HEFI-2019 and hazards of CVD 23](#_Toc109922052)

[Risks based on a fully parametric modelling of time 24](#_Toc109922053)

[Relationship between the HEFI-2019 and CVD, by sex 26](#_Toc109922054)

[Potential effect of unmeasured confounding (E-Value) 27](#_Toc109922055)

[Supplementary References 28](#_Toc109922056)

# Supplementary methods

## Estimand and hypothetical changes

The estimand of interest, i.e., the target causal effect of a hypothetical intervention, was $Pr\left( T_{A=1,C=0}>t \right)-Pr\left( T_{A=0,C=0}>t \right),\left[ 0,t \right]$. That is, the difference in probability of being CVD free during follow-up, if all participants had hypothetically “changed” their HEFI-2019 score ($A=1$) vs. if all participants had not “changed” their HEFI-2019 score ($A=0$). Both of which are estimated in a pseudo-population where lost to follow-up (i.e., 0.3% in the present study) and deaths from non-outcome causes (i.e., censoring due to competing events, 2.6%) are absent ($C=0$). Of note, the latter is a working framework to estimate the (direct) effect of a hypothetical change in the presence of competing events (1).

In the present study, the exposure of interest is adherence to the 2019 CFG recommendations on healthy food choices, which is measured on a continuous scale through the HEFI-2019. While multiple hypothetical “changes” in the HEFI-2019 scores are possible, specific scores at which there are clinically meaningful benefits in terms of CVD prevention are unknown. Thus, we have considered scenarios using plausible values based on the distribution of the HEFI-2019 score in this sample, based on usual intakes (i.e., the 5^th^, 10^th^, 25^th^, 75^th^, 90^th^ and 95^th^ percentiles). Additionally, the “no hypothetical change” condition reflect the HEFI-2019 score participants had on average in the absence of any hypothetical change, i.e., the median HEFI-2019 score (50^th^ percentile).

When $A=1$ has a percentile score *higher* than the median score (i.e., 75^th^, 90^th^ and 95^th^), the estimand corresponds to the true effect of a hypothetical intervention where all participants are successful in increasing their HEFI-2019 score up to that percentile’s score. When $A=1$ has a percentile score *lower* than the median score (i.e., 5^th^, 10^th^ and 25^th^), the corresponding interpretation of the hypothetical intervention is not as clear, because it would be unethical to intervene to reduce the HEFI-2019 and hence diet quality. However, a scenario where the HEFI-2019 is reduced has plausibility, for example, when environmental factors affect affordability or accessibility to healthy foods which, in turn, decreases the HEFI-2019 score.

## Detailed description of exclusion criteria

**Supplementary Table 1.** Detailed description of exclusion criteria ^1^

| **Exclusion criteria** | **Description** | **UK Biobank variables** |
| --- | --- | --- |
| **Cardiovascular history** | | |
| ICD-10 | Event date for I20 to I25, I48, I60 to I69 is prior to first 24-hour dietary recall | 131296, 131298, 131300, 131302, 131304, 131306, 131350, 131360, 131362, 131364, 131366, 131368, 131370, 131372, 131374, 131376, 131378 |
| Touchscreen questionnaire | Answered heart attack, angina or stroke to "Has a doctor ever told you that you have the following conditions?" | 6150 |
| Interview | Reported one or more of the following: 1471, atrial fibrillation; 1483, atrial flutter; 1074, angina; 1075, heart attack/myocardial infarction; 1081, stroke; 1082, transient ischaemic attack; 1083, subdural haemorrhage/haematoma; 1086, subarachnoid haemorrhage | 20002 |
| **Cancer history** | | |
| Touchscreen questionnaire | Answered yes to "Has a doctor ever told you that you have had cancer?" | 2453 |
| **Diabetes history** | | |
| Touchscreen questionnaire | Answered yes to "Has a doctor ever told you that you have diabetes?" | 2443 |
| **Dietary intakes** | | |
| Diet by 24-hour dietary recall | No 24-hour dietary recall completed (based on completion date) | 105010 |
| Diet by 24-hour dietary recall | Total energy intake < 418.4 KJ (100 kcal) | 100002 |
| Urine assay *^2^* | Missing value for creatinine, potassium or sodium | 30510, 30520, 30530 |
| **Missing data** | | |
| Touchscreen questionnaire | Missing values for physical activity or familial history of cardiovascular disease *^3^* | 20107, 20110, 864, 874, 884, 894, 904, 914 |

*^1^* ICD-10, International Classification of Diseases 10th revision

*^2^* Values for creatinine and potassium are required to estimate 24-h sodium intake

*^3^* Missing data for these variables was selected as an eligibility criterion because they were the most commonly missing in the otherwise eligible sample.

**Removal of 24-h dietary recalls completed after incident CVD-related events**

Data from 880 24-h dietary recalls (0.3%; covering 538 participants) were excluded from analyses because they were completed after the occurrence of a CVD event, defined using ICD-10 codes described above (**Supplementary Table 1**). This was done to avoid bias due to probable change in dietary intakes after the incident CVD.

## Source of CVD outcome data

**Supplementary Table 2.** Source of CVD outcome data ^1^

| **Source** | **Acute myocardial infarction (I21.*), n=1830** | **Cerebral infarction (I63.*), n=1013** |
| --- | --- | --- |
| Death register | 126 (6.9) | 11 (1.1) |
| Hospital admissions data | 1,423 (77.8) | 900 (88.8) |
| Primary care | 195 (10.7) | 100 (9.9) |
| Self-report | 86 (4.7) | 2 (0.2) |

^1^ Values are n (%). CVD cases (I21.* and I63.*) were identified based on the International Classification of Diseases 10^th^ revision. CVD, cardiovascular disease.

## The Healthy Eating Food Index (HEFI)-2019

The HEFI-2019 components, points and standards for scoring are described in Supplementary Table 3, adapted from (2). The food composition database of the Oxford WebQ does not provide data on sodium and free sugar intakes, which contribute to two key components of the HEFI-2019. The intake of sodium and free sugars was estimated based on data available before applying the HEFI-2019 scoring algorithm.

**Estimation of 24-h sodium intake** . The 24-h sodium intake was estimated using the predictive equation of the INTERSALT study (3) based on casual urinary sodium, potassium, and creatinine concentrations. The sex-specific equations were :

Males: $Na_{24h}=25.46+0.46\left( Na \right)-2.75\left( Cr \right)-0.13\left( K \right)+4.10\left( BMI \right)+0.26\left( Age \right)+17.05$

Females: $Na_{24h}=5.07+0.34\left( Na \right)-2.16\left( Cr \right)-0.09\left( K \right)+2.39\left( BMI \right)+2.35\left( Age \right)-0.03\left( Age^{2} \right)+12.82$

where Na is sodium (mmol/L), Cr is creatinine (mmol/L), K is potassium (mmol/L), BMI is body mass index (kg/m^2^) and age is expressed in years. The results of the equations in mmol/L were then multiplied by 23 to estimate 24-h sodium in mg. The 24-h sodium in mg was then used to apply the HEFI-2019 scoring algorithm.

**Estimation of free sugar intake.** To estimate intake of free sugars, the total sugars content of food categories rich in natural sugars was subtracted from the intake of total sugars. The food categories considered for their contribution to the intake of natural sugars were vegetables, fruits, dairy and legumes. Accordingly, sugars naturally present in honey, syrups, fruit juices and fruit juice concentrates were considered as free sugars. First, food codes from the *McCance and Widdowson* food composition table were retrieved for all single foods within the Oxford WebQ (4) as well as their serving sizes, proportion of free sugars and total sugars content. Second, the reported number of servings of all single foods was multiplied by their content in natural sugars. Third, free sugars were estimated as the difference between intake of total sugars and intake of natural sugars from vegetables, fruits, dairy foods and legumes. Based on all foods reported on all 24-h dietary recalls, the 5 main contributors to intake of natural sugars were dried fruits, prunes, banana, grapes, and peach/nectarine. Additionally, for the first 24-h dietary recall completed, mean (SD) intakes of total, natural and free sugars were 123 (62), 51 (32), 72 (51) grams/d. The ratio of mean free to mean total sugars was 0.58.

**Supplementary Table 3.** Healthy Eating Food Index (HEFI)-2019 components, points and standards for scoring

| **#** | **Component name** | **Measurement** | **Maximum Points** | **Unit** | **Standard for minimum score** | **Standard for maximum score** |
| --- | --- | --- | --- | --- | --- | --- |
| 1 | Vegetables and fruits*^1^* | Ratio: Total vegetables and fruits / Total foods*^2^* | 20 | RA/RA | No vegetables and no fruits | ≥ 0.50 |
| 2 | Whole-grain foods | Ratio: Total whole-grain foods / Total foods*^2^* | 5 | RA/RA | No whole-grain foods | ≥ 0.25 |
| 3 | Grain foods ratio*^3^* | Ratio: Total whole-grain foods / Total grain foods*^4^* | 5 | RA/RA | No whole-grain foods | = 1.0 |
| 4 | Protein foods*^5^* | Ratio: Total protein foods / Total foods*^2^* | 5 | RA/RA | No protein foods | ≥ 0.25 |
| 5 | Plant-based protein foods*^6^* | Ratio: Plant-based protein foods / Total protein foods | 5 | RA/RA | No plant-based protein foods | > 0.50 |
| 6 | Beverages | Ratio: (Plain water including carbonated + unsweetened beverages) / Total beverages*^7^* | 10 | g/g | No water and no unsweetened beverages | = 1.0 |
| 7 | Fatty acids ratio | Ratio: (Mono- + polyunsaturated fat) / Saturated fat | 5 | g/g | ≤ 1.1*^8^* | ≥ 2.6*^9^* |
| 8 | Saturated fats | Ratio: Saturated fat / energy | 5 | %E (kcal/kcal) | ≥ 15%E*^10^* | < 10%E |
| 9 | Free sugars | Ratio: Free sugars / energy | 10 | %E (kcal/kcal) | ≥ 20%E*^10^* | < 10%E |
| 10 | Sodium | Ratio: Sodium / energy | 10 | mg / kcal | ≥ 2.0 | < 0.9*^11^* |

Table adapted from Brassard et al. *Appl Physiol Nutr Metab*. 2022. CCHS, Canadian Community Health Survey; CFG-2019, Canada's food guide 2019; HEFI-2019, Healthy Eating Food Index 2019; RA, Reference Amounts (amount of food usually eaten by an individual at one sitting); %E, percent of total energy.

*^1^* All vegetables and fruits regardless of saturated fat, sodium or free sugar content; excludes fruit juice (i.e., considered as sugary drinks in CFG-2019).

*^2^* Total foods include all foods consumed as well as beverages considered in protein foods (i.e., unsweetened milk and unsweetened plant-based beverages that contain protein); excludes all other beverages as well as solid fats, oils and spreads and culinary ingredients (e.g., spices and baking soda).

*^3^* Foods where the first ingredient is either whole grains or whole wheat, regardless of saturated fat, sodium or free sugar content.

*^4^* Foods where the first ingredient is a grain (whole or not) regardless of saturated fat, sodium or free sugar content.

*^5^* All protein foods regardless of fat, sodium or sugars content; excludes processed meats (i.e., not considered protein foods in CFG-2019) and sweetened milks (i.e., considered as sugary drinks in CFG-2019).

*^6^* All plant-based protein foods, regardless of saturated fat, sodium or free sugar content.

*^7^* Unsweetened beverages include unsweetened coffee and tea, unsweetened milk and unsweetened plant-based beverages. Total beverages include water (plain or carbonated), coffee, tea, milk and plant-based beverages, fruit and vegetable juices, alcoholic drinks, artificially sweetened beverages and sugary drinks.

*^8^* Approximately the **15th percentile** of intake based on data (single 24-h dietary recall) in Canadians from the 2015 CCHS – Nutrition.

*^9^* Corresponds to the **1st percentile** of unsaturated to saturated fats ratios among simulated diets developed to be fully consistent with all recommendations in CFG-2019.

*^10^* Approximately the **85th percentile** of intake based on data (single 24-h dietary recall) in Canadians from the 2015 CCHS – Nutrition.

*^11^* Standard for maximum points based on the Chronic Disease Risk Reduction for 14+ years (i.e., 2300 mg) over the **90th percentile** of usual energy intakes in respondents 2 y and older from the 2015 CCHS – Nutrition (i.e., approximately 2600 kcal).

## Classification of foods in the Oxford WebQ


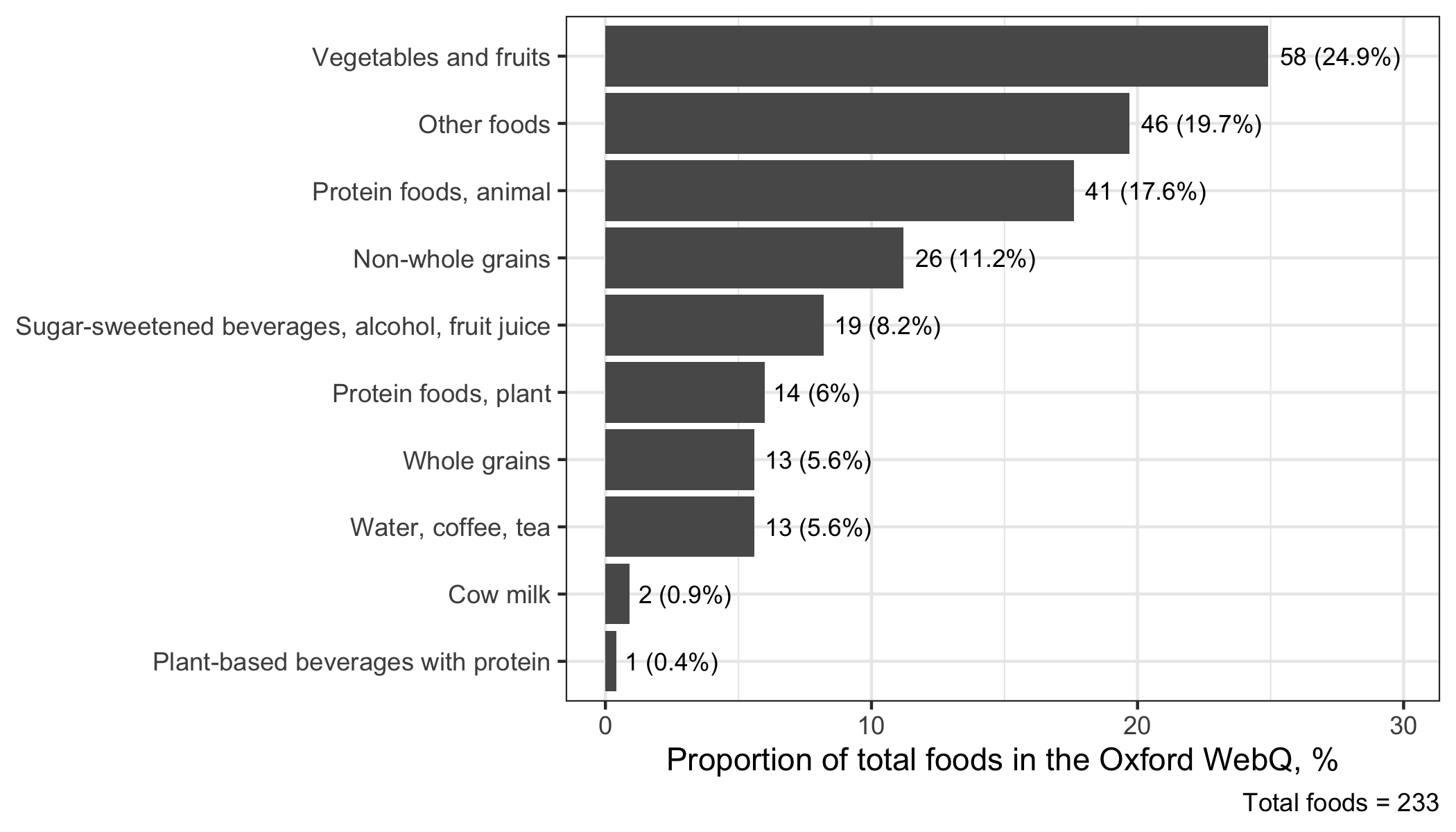


**Supplementary Figure 1.** Distribution of unique foods in the Oxford WebQ according to the HEFI-2019 food categories. Foods that are mainly fats (e.g., oils and spreads) are considered as nutrients in the HEFI-2019 (not shown). HEFI-2019, Healthy Eating Food Index 2019

## Covariates

**Supplementary Table 4** presents the covariates and their functional forms used in the inverse probability weighting models or in the Cox regression models (HEFI-2019 total score and energy intake only). All covariates assessed on a continuous scale were *a priori* transformed with a restricted cubic spline function to address plausible nonlinearity.

**Supplementary Table 4.** Covariate names, UK Biobank codes and functional forms ^1^

| **No** | **Variable name** | **UK Biobank code** | **Functional form** | **Missing data, n (%)** *^2^* |
| --- | --- | --- | --- | --- |
| 1 | Sex | 31 | 2 categories | - |
| 2 | Age *^3^* | 34, 52, 105010 | Restricted cubic spline (5 knots) | - |
| 3 | Region | 54 | 3 categories | - |
| 4 | Townsend deprivation index | 189 | Restricted cubic spline (3 knots) | 169 (0.12%) |
| 5 | University degree | 6138 | 2 categories | 382 (0.28%) |
| 6 | Employment | 6142 | 3 categories | 234 (0.17%) |
| 7 | Familial history of cardiovascular disease | 20107, 20110 | 3 categories | - |
| 8 | Menopausal status (female only) | 2724 | 3 categories | 51 (0.04%) |
| 9 | Hormone replacement use (female only) | 2814 | 2 categories | 136 (0.10%) |
| 10 | Smoking habits | 20116 | 3 categories | 202 (0.15%) |
| 11 | Physical activity level | 864, 874, 884, 894, 904, 914 | 3 categories | - |
| 12 | Alcohol consumption habits | 20117 | 3 categories | 50 (0.04%) |
| 13 | Sedentary time (television, computer, driving) | 1070, 1080, 1090 | Restricted cubic spline (4 knots) | 8 (0.01%) |
| 14 | Body mass index | 21001 | Restricted cubic spline (5 knots) | 225 (0.16%) |
| 15 | Dietary supplement use *^4^* | 104670 | 2 categories | - |
| 16 | Medication (cholesterol and/or blood pressure) | 6153, 6177 | 3 categories | 474 (0.35%) |
| 17 | Risk factor (high cholesterol and/or high blood pressure) | 6150, 20002 | 3 categories | 1 (0.00%) |
| 18 | HEFI-2019 total score *^4^* | NA | Restricted cubic spline (4 knots) | - |
| 19 | Energy intake *^4^* | 100002 | Restricted cubic spline (4 knots) | - |

*^1^* All variables were measured at the baseline visit, unless otherwise indicated. HEFI-2019, Healthy Eating Food Index-2019. NA, not applicable

*^2^* A total of 1.5% of data was missing in the included sample, which were imputed once by chained equations. We used a discriminant function for unordered categorical variables, logistic for binary or ordered categorical variables and predictive mean matching for continuous variables.

*^3^* Age was calculated as the age at the first 24-h dietary recall completion.

*^4^* Assessed at the online follow-up (i.e., completion of the 24-h dietary recall).

## Plausibility of self-reported energy intakes

Self-reported energy intakes of all 24-h dietary recalls were compared with predicted energy requirements to assess their plausibility (5). The physical activity level in predictive energy requirements equations was assumed to be sedentary for all participants. Cut-offs to define plausible energy intakes were calculated as follows:

$$\pm1SD=\sqrt{\left( CV_{rEI}^{2}/d+CV_{pER}^{2}+CV_{mTEE}^{2} \right)}$$

where $CV_{rEI}^{2}$ is the within-individual coefficient of variation of self-reported energy intake, $d$ is the number of 24-h dietary recalls completed, $CV_{pER}^{2}$ is the between-individual coefficient of variation of predicted energy requirements and $CV_{mTEE}^{2}$ is the coefficient of variation in measured total energy expenditure (mTEE; from (6)). The resulting cut-offs used to assess the plausibility of reported energy intakes are presented in Supplementary Table 5. For example, among all participants, and based on a SD of 26%, participants with a self-reported energy intake to predicted energy requirement between 0.74 and 1.26 were considered to have plausible energy intake data.

**Supplementary Table 5.** Estimation of cut-off to determine the plausibility of self-reported energy intakes in adults from the UK Biobank, by BMI, sex and age categories ^1^

| **Stratum** | **Sample size, n** | **24-h recall completed, n** | **Coefficient of variation** | | | **±1 SD cut-off** |
| --- | --- | --- | --- | --- | --- | --- |
|  |  |  | Self-reported energy intake | Predicted energy requirements | Biological variability*^2^* |  |
| **All** | 136,698 | 2.2 | 26.2% | 17.0% | 8.2% | 26% |
| **BMI <25.0 kg/m² - Males** | | | | | | |
| 40 to 50 y | 4,930 | 2.2 | 26.1% | 6.3% | 8.2% | 20% |
| 51 to 70 y | 13,552 | 2.3 | 23.9% | 6.9% | 8.2% | 19% |
| 71 y+ | 450 | 2.1 | 22.6% | 6.1% | 8.2% | 19% |
| **BMI <25.0 kg/m² - Females** | | | | | | |
| 40 to 50 y | 10,452 | 2.3 | 26.4% | 5.4% | 8.2% | 20% |
| 51 to 70 y | 25,223 | 2.3 | 24.6% | 6.1% | 8.2% | 19% |
| 71 y+ | 531 | 2.0 | 22.7% | 5.4% | 8.2% | 19% |
| **BMI ≥25.0 kg/m² - Males** | | | | | | |
| 40 to 50 y | 10,184 | 2.0 | 29.4% | 7.3% | 8.2% | 23% |
| 51 to 70 y | 31,053 | 2.2 | 26.0% | 7.9% | 8.2% | 21% |
| 71 y+ | 928 | 2.1 | 25.1% | 6.9% | 8.2% | 21% |
| **BMI ≥25.0 kg/m² - Females** | | | | | | |
| 40 to 50 y | 9,383 | 2.1 | 29.6% | 8.9% | 8.2% | 24% |
| 51 to 70 y | 29,332 | 2.2 | 26.8% | 8.8% | 8.2% | 22% |
| 71 y+ | 680 | 1.9 | 24.1% | 7.6% | 8.2% | 21% |

*^1^* The within-individual coefficients of variation for total energy intakes measured using the 24-h dietary recalls was determined using the National Cancer Institute univariate method and log-transformed data. BMI, body mass index.

*^2^* The normal day-to-day variation in objectively measured total energy expenditure was obtained from Black & Cole, Eur J Clin Nutr 2000.

## Statistical analyses

### **Steps 1 and 2: Modelling of dietary constituents.**

Dietary constituents of the HEFI-2019 were modelled using the National Cancer Institute (NCI) multivariate method (7) to mitigate within-individual random errors (8). This method estimates usual (long-term) intakes hence mitigating to a large extent the influence of any given 24-h recall reflecting days with very low or very high energy intakes. From our experience, 24-h recall with zero energy intake cannot effectively contribute to estimating usual intakes based on the NCI method. However, it is hard to draw the line at which a given energy level in a 24-h recall is not enough in a large study like the UK Biobank with many repeated 24-h dietary calls (up to 5) and many participants (>130,000). 24-h dietary recall data were included in the NCI modeling of usual intakes if the reported energy intake was 100 kcal or more, an arbitrary cut-off. As few as 114 recalls were excluded due to reported energy intake below 100 kcal on a total of over 450,000 24-h dietary recalls completed. Once modelled to consider random errors, the estimated 1^st^ percentile of (modelled) usual energy intakes was approximately 1260 kcal. Finally, under-reporting of energy intake, approximately 14% of participants in the eligible sample, is not higher than previously found in nutrition survey (Supplementary Table 11).

First, the multivariate method was used to derive distribution of HEFI-2019 scores, based on usual intakes. In this first model, the covariates were indicators of sequence of 24-h dietary recall (second, third, fourth or fifth), indicator of weekend 24-h dietary recall (Friday, Saturday or Sunday), age groups (<51 y, 51 to < 71 y, 71 y or older) and an indicator of CVD outcome or death censoring during follow-up. The measurement error model was stratified by sex. Refined-grain foods, whole-grain foods, plant-based protein foods, unsweetened plant-based beverages and beverages not recommended in the 2019 CFG (i.e., sugary drinks, artificially sweetened beverages, fruit juices, sweetened milk and plant-based beverages, alcohol) were considered as episodically consumed in the multivariate method, while the remaining foods and nutrients were considered as daily consumed. A total of 500 simulations were performed in the Monte Carlo simulation step of the multivariate method. Simulations from both strata (i.e., males and females) were combined before estimating distributions.

Second, the multivariate method was used to simulate usual intakes at the participant level to obtain measurement-error-corrected regression coefficients in diet-outcome models (7). The covariates were those used to mitigate confounding (i.e., sex, age, region, Townsend deprivation index, university degree, employment, familial history of cardiovascular disease, menopausal status (female only), hormone replacement use (female only), smoking habits, physical activity level, alcohol consumption habits, sedentary time, body mass index, dietary supplement use, medication use, and self-reported risk factor (high cholesterol and/or high blood pressure)) and the predicted 24-h sodium intake based on the urine assay. The dietary constituents were modelled as described above, except for unsweetened plant-based beverages, which had to be added to larger food categories (plant-based protein foods and unsweetened beverages) due to model non-convergence. A total of 1,000 simulations (pseudo-individuals) were performed in the Monte Carlo simulation step of the multivariate method. The total HEFI-2019 score was calculated among pseudo-individuals after applying the HEFI-2019 scoring algorithm. The total HEFI-2019 score and energy intake were both transformed *a priori* with a restricted cubic spline function including 4 knots (i.e., the 5^th^, 35^th^, 65^th^ and 95^th^ percentile of the score distribution based on usual intakes) using a SAS macro by Desquilbet and Mariotti (9). Simulated usual intakes and derived variables were then averaged across the 1,000 pseudo-individuals before further analysis.

### **Step 3: Adjustments using inverse probability weighting (IPW)**

The use of IPW instead of more traditional covariate adjustment was justified by the simplicity of making adjusted survival curves (10), to mitigate the potential bias due to adjustments for variables that may not always be a confounder (e.g., body mass index; (11)) and to account for informative censoring (1,11). The inverse probability of “treatment” weights (IPTW) were estimated via a linear regression model including all covariates and assuming a normal distribution for the HEFI-2019 (12,13). Informative censoring due to mortality (i.e., competing events) was accounted with inverse probability of censoring weighting (IPCW) estimated via logistic regression. The IPTW and IPCW weights were both stabilized to the sample size and then multiplied to obtain combined weights for analysis. The combined (stabilized) weights equation is:

$${SW}^{X,C}={SW}^{X}\times{SW}^{C}=\frac{f\left( X_{0} \right)}{f\left( X_{0} | L_{0} \right)}\times\frac{\Pr\left[ C=0 | X_{0} \right]}{\Pr\left[ C=0 | X_{0},E_{0}, L_{0} \right]}$$

Where ${SW}^{X,C}$are combined weights; ${SW}^{X}$ are stabilized IPTW; ${SW}^{C}$ are stabilized IPCW; $X_{0}$ is the total HEFI-2019 score measured at the start of follow-up; $E_{0}$ is energy intake measured at the start of follow-up; $L_{0}$ is a vector of covariates measured at the start of follow-up (i.e., confounders); and C is an indicator variable for any censoring during the follow-up. $f\left( \cdot\right)$ for ${SW}^{X}$ is a probability density function assuming normal (Gaussian) density. The denominator and the numerator of both IPTW and IPCW are estimated in separate models. For IPTW (${SW}^{X})$, the denominator is estimated using a linear regression model of the HEFI-2019 score on covariates. Accordingly, $f\left( X_{0} | L_{0} \right)$ is a probability density function assuming normal (Gaussian) density with mean $\mu_{L_{0}}=f\left( X_{0} | L_{0} \right)$ and constant variance $\sigma_{DEN}^{2}$ (13). The numerator is also estimated using a linear regression model and $f\left( X_{0} \right)$ is also a probability density function assuming normal (Gaussian) density with mean $\mu=f\left( X_{0} \right)$ and constant variance $\sigma_{NUM}^{2}$. For IPCW (${SW}^{C}$), the denominator is estimated using a logistic regression model of the censoring indicator on the HEFI-2019 score, energy intake and all covariates. The logistic regression model predicted probabilities are then generated to obtain the probability of remaining in the study for each participant (i.e., uncensored $(\Pr\left[ C=0 | X_{0},E_{0}, L_{0} \right])$).

Under the assumption that there is no unmeasured confounders, that models for estimating weights are correctly specified and that there was no measurement error, applying the combined weights to the study participants yields a pseudo-population in which the HEFI-2019 score distribution is independent of confounders (12,14) and without informative censoring (i.e., competing events; (1)). However, the assumption of no unmeasured confounders can never be fully verified as in all observational studies. Whether this assumption is satisfied depends on the extent to which all relevant variables were considered to mitigate confounding (**Supplementary Table 4**). When estimating IPW, the use of flexible modelling strategies for continuous covariates is recommended to consider nonlinear relationships (15). Restricted cubic spline transformations were used in the present study, which should contribute to better covariate balance and to mitigate bias (15). Dietary intakes measured with the 24-h dietary recall instrument are mostly affected by random measurement errors. However, the NCI multivariate method was used to account for these random errors (7), which could otherwise cause bias (16).

The **Supplementary Figure 2** presents the relationship between the total HEFI-2019 score and confounders with and without weighting by the inverse probability of treatment weights (${SW}^{X})$. As expected, weighting importantly attenuated the standardized regression coefficients between the total HEFI-2019 score and confounders.

**Supplementary Figure 2.** Standardized regression coefficients from the linear regression of the total HEFI-2019 score on baseline covariate, without weighting (None) and with weighting (Normal). Reference level of categorical variables are omitted. BMI, body mass index; Dietsuppl, use of dietary supplement; Employ, employment situation; Famhist, familial history of cardiovascular disease; Hrt, hormone replacement therapy; Meno, menopausal status; Physact, physical activity; Roh, alcohol habits; Smk, smoking status.

### **Step 4: outcome model for incident CVD**

The Cox proportional hazards regression model was used to analyze the relationship between the total HEFI-2019 score based on usual intakes and incident CVD. Accordingly, the outcome was modelled as:

$$\lambda\left( t|X_{0},E_{0} \right)=\lambda_{0}\left( t \right)\cdot exp\left( \beta_{X_{0}}X_{0}+\beta_{E_{0}}E_{0} \right)$$

However, contrasts in counterfactual hazards generally cannot have a causal interpretation (1, 17). Thus, the Cox regression parameter estimates from the outcome model were used to generate survival curves reflecting each predetermined percentile of the HEFI-2019 scores distribution determined in step 1. In other words, the Cox regression model was used to estimate the cumulative survival probability had all participants had a HEFI-2019 score at the predetermined percentile value and at constant energy intake. To derive the survival curves at predetermined HEFI-2019 score percentiles, we substituted $X_{0}$ with the predetermined HEFI-2019 score in:

$$S\left( t \right)=\left[ S_{0}\left( t \right) \right]^{\exp\left( \beta_{X_{0}}X_{0}+\beta_{E_{0}}E_{0} \right)}$$

For energy intake at the start of follow-up ($E_{0})$, the mean energy intake in this sample was used for all survival curves (i.e., 2100 kcal), ensuring that energy intake remains constant across all curves. Concretely, the survival curve at the end of follow-up (137 months) at the 90th percentile of the HEFI-2019 score distribution (58.1 points) is obtained with:

$$S\left( t_{137} \right)=\left[ S_{0}\left( t \right) \right]^{\exp\left( \beta_{X_{0}}\cdot58.1+\beta_{E_{0}}\cdot2100 \right)}$$

Risks are then calculated as $1-S\left( t_{137} \right)$. Finally, contrasts in counterfactual risks are derived by comparing risks at predetermined percentile of the HEFI-2019 scores distribution. Of note, restricted cubic spline transformations for the HEFI-2019 and total energy intake are not shown in the equations above.

# Supplementary results

## Characteristics of participants

**Supplementary Table 6.** Baseline characteristics of UK Biobank participants, by inclusion status in the present study^1^

| **Characteristics** | **Excluded, n=365,761** | **Included, n=136,698** |
| --- | --- | --- |
| **Age at baseline assessment, y** | | |
| Mean (SD) | 57.6 (8.09) | 55.6 (7.91) |
| 55 y or younger | 131,695 (36.0%) | 61,837 (45.2%) |
| 55 to <65 y | 154,702 (42.3%) | 57,344 (41.9%) |
| 65 y or older | 79,364 (21.7%) | 17,517 (12.8%) |
| **Sedentary time, h/d** | | |
| Mean (SD) | 4.88 (2.51) | 4.61 (2.30) |
| **Body mass index** | | |
| Mean (SD) | 27.8 (4.91) | 26.5 (4.38) |
| Underweight, <18.5 | 1,832 (0.5%) | 794 (0.6%) |
| Normal, 18.5-24.9 | 108,171 (29.8%) | 54,222 (39.7%) |
| Overweight, 25-29.9 | 155,305 (42.8%) | 56,793 (41.6%) |
| Obese, >29.9 | 97,574 (26.9%) | 24,664 (18.1%) |
| **Sex** | | |
| Females | 197,752 (54.1%) | 75,601 (55.3%) |
| Males | 168,009 (45.9%) | 61,097 (44.7%) |
| **Region** | | |
| England | 320,144 (87.5%) | 125,670 (91.9%) |
| Wales | 16,812 (4.6%) | 3,994 (2.9%) |
| Scotland | 28,805 (7.9%) | 7,034 (5.1%) |
| **White/british ethnic background** | | |
| Yes | 321,031 (88.3%) | 122,089 (89.5%) |
| **Familial history of cardiovascular disease** | | |
| None | 130,435 (42.1%) | 61,379 (44.9%) |
| Father`s or mother`s side | 133,987 (43.2%) | 57,658 (42.2%) |
| Both | 45,513 (14.7%) | 17,661 (12.9%) |
| **Education level** | | |
| No diploma | 76,724 (21.6%) | 8,541 (6.3%) |
| Vocational qualification (NVQ or HND or HNC) | 52,377 (14.7%) | 18,173 (13.3%) |
| Any school degree (A, AS, O, GCSE, CSE) | 99,522 (28.0%) | 40,837 (30.0%) |
| College, university degree or professional qualification | 127,390 (35.8%) | 68,765 (50.4%) |
| **Employment situation** | | |
| Working | 197,423 (54.4%) | 89,694 (65.7%) |
| Retired | 129,442 (35.7%) | 37,533 (27.5%) |
| Other | 36,180 (10.0%) | 9,237 (6.8%) |
| **Townsend deprivation index** | | |
| T1 (min , -3.1) | 117,550 (32.2%) | 49,813 (36.5%) |
| T2 (>-3.1 , -0.6) | 120,032 (32.9%) | 47,166 (34.5%) |
| T3 (>-0.6, max) | 127,725 (35.0%) | 39,550 (29.0%) |
| **Alcohol consumption habits** | | |
| Never | 18,405 (5.1%) | 3,979 (2.9%) |
| Previous | 14,525 (4.0%) | 3,574 (2.6%) |
| Current | 331,229 (91.0%) | 129,095 (94.5%) |
| **Smoking habits** | | |
| Never | 193,436 (53.3%) | 80,060 (58.7%) |
| Previous | 126,860 (34.9%) | 46,184 (33.8%) |
| Current | 42,719 (11.8%) | 10,252 (7.5%) |
| **Physical activity level** | | |
| Low | 40,031 (14.5%) | 18,325 (13.4%) |
| Moderate | 129,949 (47.0%) | 66,294 (48.5%) |
| High | 106,797 (38.6%) | 52,079 (38.1%) |
| **Major dietary habits change in the past 5 years** | | |
| None | 214,550 (59.0%) | 88,904 (65.1%) |
| Yes, because of other reasons | 100,466 (27.6%) | 40,654 (29.8%) |
| Yes, because of illness | 48,450 (13.3%) | 7,072 (5.2%) |
| **Dietary supplement use** | | |
| Yes | 36,249 (48.8%) | 67,585 (49.4%) |
| **Medication use** | | |
| None | 243,765 (68.2%) | 112,663 (82.7%) |
| Cholesterol- or blood pressure-lowering | 66,572 (18.6%) | 17,407 (12.8%) |
| Both | 47,296 (13.2%) | 6,154 (4.5%) |
| **Menopausal status (female only)** | | |
| Not sure | 32,467 (16.5%) | 10,428 (13.8%) |
| Yes | 122,874 (62.4%) | 42,516 (56.3%) |
| **Hormone replacement therapy (female only)** | | |
| Yes | 78,838 (40.2%) | 25,069 (33.2%) |
| **Cardiovascular disease risk factor** | | |
| None | 238,072 (65.3%) | 104,251 (76.3%) |
| High cholesterol or blood pressure | 102,219 (28.0%) | 27,079 (19.8%) |
| Both | 24,542 (6.7%) | 5,367 (3.9%) |

*^1^* Values are mean (SD) or n (%). Percentages are column percentage and reflect the proportion of participants within groups (i.e., included or excluded). Frequencies may not sum to the total number of participants due to missing data.

## Dietary intakes

**Supplementary Table 7.** Estimated mean usual intakes of foods and nutrients in adults from the UK Biobank^1^

| **Dietary constituents** | **All, n=136,698** | **Quarters of HEFI-2019 total score** | | | |
| --- | --- | --- | --- | --- | --- |
|  |  | Q1 (min - 39.5) | Q2 (>39.5 - 46.5) | Q3 (>46.5 - 53) | Q4 (>53- max) |
| Vegetables and fruits, servings/d | 5.9 (2.8) | 3.6 (1.5) | 5.1 (1.8) | 6.4 (2.1) | 8.6 (2.6) |
| Whole-grain foods, servings/d | 1.9 (1.1) | 1.3 (1.0) | 1.8 (1.0) | 2.0 (1.1) | 2.3 (1.0) |
| Non-whole grain foods, servings/d | 1.8 (1.0) | 2.4 (1.0) | 1.9 (0.9) | 1.7 (0.8) | 1.4 (0.7) |
| Protein, animal-based, servings/d | 2.5 (0.9) | 2.6 (0.9) | 2.6 (0.9) | 2.5 (0.8) | 2.5 (0.8) |
| Protein, plant-based, servings/d | 0.4 (0.4) | 0.3 (0.3) | 0.4 (0.3) | 0.4 (0.3) | 0.6 (0.4) |
| Other low nutritive value foods, servings/d | 4.1 (3.1) | 6.5 (4.0) | 4.3 (2.6) | 3.3 (2.0) | 2.4 (1.5) |
| Water, coffee and tea, ml/d | 1,393 (412) | 1,201 (375) | 1,343 (381) | 1,441 (389) | 1,586 (403) |
| Milk, ml/d | 211 (142) | 227 (147) | 220 (146) | 208 (140) | 188 (130) |
| Soy beverage, ml/d | 5.2 (23.3) | 1.9 (12.0) | 3.5 (17.8) | 5.2 (22.7) | 10.2 (34.1) |
| SSBs, alcohol and fruit juice, ml/d | 569 (401) | 732 (461) | 600 (401) | 521 (359) | 424 (300) |
| MUFA, g | 33 (9) | 35 (9) | 34 (9) | 33 (9) | 32 (8) |
| PUFA, g | 14 (4) | 14 (4) | 14 (4) | 14 (4) | 14 (4) |
| SFA, g | 30 (9) | 34 (10) | 31 (9) | 28 (8) | 26 (7) |
| SFA, %E | 12.5 (2.4) | 13.8 (2.3) | 12.9 (2.2) | 12.1 (2.1) | 11.0 (1.9) |
| Free sugars, g *^2^* | 72 (30) | 91 (32) | 75 (28) | 65 (25) | 56 (21) |
| Free sugars, %E *^2^* | 13.3 (4.1) | 16.5 (4.0) | 13.8 (3.6) | 12.3 (3.3) | 10.6 (2.8) |
| Predicted 24-h sodium, mg *^3^* | 3,238 (777) | 3,682 (756) | 3,351 (741) | 3,123 (702) | 2,798 (619) |
| Energy, kcal | 2,128 (457) | 2,193 (468) | 2,150 (470) | 2,101 (457) | 2,069 (424) |

*^1^* Values are mean (SD). All data except predicted 24-h sodium were modeled using the National Cancer Institute's multivariate method (see Methods) to estimate usual intakes.

*^2^* Free sugars were estimated as the difference between total sugars intake and the calculated natural sugars contribution from vegetables, fruits, dairy foods and legumes.

*^3^* 24-h sodium intakes were estimated based on (spot) sodium in urine assay. Sex-specific INTERSALT equations (Western Europe) were used to predict 24-h sodium (Brown et al. American Journal of Epidemiology 2013).

**Supplementary Table 8.** Estimated means of Healthy Eating Food Index (HEFI)-2019 component and total scores in adults from the UK Biobank^1^

| **HEFI-2019 components** | **All, n=136,698** | **Quarters of HEFI-2019 total score** | | | |
| --- | --- | --- | --- | --- | --- |
|  |  | Q1 (min - 39.5) | Q2 (>39.5 - 46.5) | Q3 (>46.5 - 53) | Q4 (>53- max) |
| Vegetables and fruits (/20) | 13.2 (4.5) | 8.3 (3.0) | 12.0 (3.0) | 14.8 (3.0) | 17.7 (2.4) |
| Whole-grain foods (/5) | 2.1 (1.1) | 1.5 (1.1) | 2.1 (1.1) | 2.4 (1.1) | 2.5 (1.0) |
| Grain foods ratio (/5) | 2.5 (1.1) | 1.8 (1.1) | 2.4 (1.0) | 2.7 (1.0) | 3.1 (0.9) |
| Protein foods (/5) | 4.2 (0.8) | 4.1 (0.9) | 4.3 (0.8) | 4.3 (0.7) | 4.2 (0.7) |
| Plant-based protein foods (/5) | 1.2 (1.0) | 0.8 (0.7) | 1.0 (0.9) | 1.3 (0.9) | 1.7 (1.1) |
| Beverages (/10) | 7.5 (1.4) | 6.7 (1.5) | 7.3 (1.4) | 7.7 (1.3) | 8.1 (1.1) |
| Fatty acids ratio (/5) | 1.8 (1.0) | 1.3 (0.8) | 1.6 (0.9) | 1.9 (0.9) | 2.5 (1.0) |
| Saturated fats (/5) | 2.6 (1.8) | 1.6 (1.6) | 2.3 (1.7) | 2.8 (1.6) | 3.7 (1.4) |
| Free sugars (/10) *^2^* | 6.5 (3.2) | 3.9 (3.1) | 6.1 (3.0) | 7.3 (2.6) | 8.6 (1.9) |
| Sodium (/10) *^3^* | 4.3 (3.0) | 3.1 (2.8) | 4.0 (2.9) | 4.6 (2.9) | 5.6 (2.8) |
| Total score (/80) | 46.0 (9.6) | 33.2 (4.9) | 43.2 (2.0) | 49.7 (1.9) | 57.8 (3.5) |

*^1^* Values are mean (SD) scores. The HEFI-2019 was calculated based on usual dietary intakes collected using 24-h dietary recalls (except sodium) and modeled using the National Cancer Institute's multivariate method (see Methods).

*^2^* Free sugars were estimated as the difference between total sugars intake and the calculated natural sugars contribution from vegetables, fruits, dairy foods and legumes.

*^3^* 24-h sodium intakes were estimated based on (spot) sodium in urine assay. Sex-specific INTERSALT equations (Western Europe) were used to predict 24-h sodium (Brown et al. American Journal of Epidemiology 2013).

**Supplementary Table 9.** Estimated percentiles of Healthy Eating Food Index (HEFI)-2019 component and total scores in 136,698 adults from the UK Biobank^1^

| **Components** | **Percentiles** | | | | | | | | |
| --- | --- | --- | --- | --- | --- | --- | --- | --- | --- |
|  | 1 | 5 | 10 | 25 | 50 | 75 | 90 | 95 | 99 |
| Vegetables and fruits (/20) | 3.4 | 5.6 | 7.1 | 9.9 | 13.3 | 16.9 | 19.9 | 20.0 | 20.0 |
| Whole-grain foods (/5) | 0.1 | 0.4 | 0.7 | 1.3 | 2.1 | 2.9 | 3.7 | 4.2 | 5.0 |
| Grain foods ratio (/5) | 0.1 | 0.5 | 0.9 | 1.7 | 2.6 | 3.3 | 3.9 | 4.2 | 4.6 |
| Protein foods (/5) | 2.2 | 2.8 | 3.1 | 3.7 | 4.4 | 5.0 | 5.0 | 5.0 | 5.0 |
| Plant-based protein foods (/5) | 0.0 | 0.1 | 0.2 | 0.4 | 0.9 | 1.7 | 2.6 | 3.2 | 4.3 |
| Beverages (/10) | 3.6 | 4.8 | 5.5 | 6.6 | 7.6 | 8.5 | 9.2 | 9.5 | 9.9 |
| Fatty acids ratio (/5) | 0.0 | 0.4 | 0.6 | 1.1 | 1.7 | 2.4 | 3.2 | 3.7 | 4.8 |
| Saturated fats (/5) | 0.0 | 0.0 | 0.0 | 1.0 | 2.7 | 4.2 | 5.0 | 5.0 | 5.0 |
| Free sugars (/10) *^2^* | 0.0 | 0.0 | 1.3 | 4.2 | 7.1 | 9.6 | 10.0 | 10.0 | 10.0 |
| Sodium (/10) *^3^* | 0.0 | 0.0 | 0.0 | 1.7 | 4.4 | 6.7 | 8.4 | 9.3 | 10.0 |
| Total score (/80) | 22.7 | 29.3 | 33.0 | 39.5 | 46.5 | 53.0 | 58.1 | 60.7 | 64.9 |

*^1^* Values are percentile of the distribution. Percentile values in this table may differ slightly from percentile values of the outcome model, since values of the latter excludes censored participants (n=3,530; 2.6%) while the former includes them. The HEFI-2019 was calculated based on usual dietary intakes modeled using the National Cancer Institute's multivariate method (see Methods).

*^2^* Free sugars were estimated as the difference between total sugars intake and the calculated natural sugars contribution from vegetables, fruits, dairy foods and legumes.

*^3^* 24-h sodium intakes were estimated based on (spot) sodium in urine assay. Sex-specific INTERSALT equations (Western Europe) were used to predict 24-h sodium (Brown et al. American Journal of Epidemiology 2013).

**Supplementary Table 10.** Estimated Pearson correlation coefficients of HEFI-2019 component and total scores in 136,698 adults from the UK Biobank^1^

| **Component** | **Vegetables and fruits** | **Whole-grain foods** | **Grain foods ratio** | **Protein foods** | **Plant-based protein foods** | **Beverages** | **Fatty acids ratio** | **Saturated fats** | **Free sugars** | **Sodium** |
| --- | --- | --- | --- | --- | --- | --- | --- | --- | --- | --- |
| Vegetables and fruits | 1.00 | - | - | - | - | - | - | - | - | - |
| Whole-grain foods | 0.05 | 1.00 | - | - | - | - | - | - | - | - |
| Grain foods ratio | 0.28 | 0.83 | 1.00 | - | - | - | - | - | - | - |
| Protein foods | −0.10 | 0.01 | −0.06 | 1.00 | - | - | - | - | - | - |
| Plant-based protein foods | 0.25 | 0.08 | 0.10 | −0.18 | 1.00 | - | - | - | - | - |
| Beverages | 0.23 | 0.07 | 0.15 | −0.08 | 0.05 | 1.00 | - | - | - | - |
| Fatty acids ratio | 0.28 | 0.12 | 0.14 | −0.07 | 0.40 | 0.04 | 1.00 | - | - | - |
| Saturated fats | 0.39 | 0.15 | 0.18 | −0.12 | 0.21 | −0.15 | 0.66 | 1.00 | - | - |
| Free sugars *^2^* | 0.33 | 0.11 | 0.09 | 0.28 | 0.05 | 0.32 | 0.19 | 0.02 | 1.00 | - |
| Sodium *^3^* | 0.12 | 0.03 | 0.12 | −0.13 | 0.11 | 0.06 | −0.04 | −0.08 | −0.14 | 1.00 |
| Residual HEFI-2019 *^4^* | 0.50 | 0.24 | 0.39 | −0.06 | 0.27 | 0.25 | 0.39 | 0.28 | 0.28 | 0.02 |

*^1^* The HEFI-2019 was calculated based on dietary intakes modeled using the National Cancer Institute's multivariate method (see Methods). HEFI-2019, Healthy Eating Food Index 2019.

*^2^* Free sugars were estimated as the difference between total sugars intake and the calculated natural sugars contribution from vegetables, fruits, dairy foods and legumes.

*^3^* 24-h sodium intakes were estimated based on (spot) sodium in urine assay. Sex-specific INTERSALT equations (Western Europe) were used to predict 24-h sodium (Brown et al. American Journal of Epidemiology 2013).

*^4^* For the correlation between a given component score and the (residual) HEFI-2019, the HEFI-2019 corresponded to the total HEFI-2019 from which points from the component being assessed were subtracted.

**Supplementary Table 11.** Plausibility of self-reported energy intakes in 136,698 adults from the UK Biobank, by age, sex and body mass index *^1^*

|  | **Under-reporting** | **Plausible reporting** | **Over-reporting** |
| --- | --- | --- | --- |
| **All** | 19,966 (14.6%) | 89,308 (65.3%) | 27,424 (20.1%) |
| **Sex** | | | |
| Females | 7,887 (10.4%) | 48,480 (64.1%) | 19,234 (25.4%) |
| Males | 12,079 (19.8%) | 40,828 (66.8%) | 8,190 (13.4%) |
| **Age** | | | |
| 55 y or younger | 9,423 (17.7%) | 34,571 (64.8%) | 9,333 (17.5%) |
| 55 to <65 y | 7,819 (13.7%) | 37,970 (66.3%) | 11,487 (20.1%) |
| 65 y or older | 2,724 (10.4%) | 16,767 (64.3%) | 6,604 (25.3%) |
| **Body mass index, kg/m²** | | | |
| Below 30 | 13,598 (12.2%) | 73,794 (66.0%) | 24,417 (21.8%) |
| 30 or above | 6,316 (25.6%) | 15,385 (62.4%) | 2,963 (12.0%) |

*^1^* Values are n (%). The percentages add up to 100% among columns. The plausibility of reported energy intakes was estimated using the method by Huang et al. Plausible reporting corresponded to a ratio of reported energy intake to predicted energy requirements within 0.74 and 1.26. See Supplementary Methods for details.

## Relationship between the HEFI-2019 and hazards of CVD

**Supplementary Table 12.** Hazard ratios for CVD according to varying length of follow-up in 132,777 adults from the UK Biobank ^1^

| **Total HEFI-2019 score** | | **Varying length of follow-up** | | | | 11 years (full) |
| --- | --- | --- | --- | --- | --- | --- |
| Percentile | Points (/80) | 1 year | 3 years | 5 years | 7 years |  |
| 95 | 60.7 | 0.46 (0.10,0.83) | 0.94 (0.30,1.59) | 0.64 (0.29,1.00) | 0.62 (0.35,0.88) | 0.68 (0.43,0.93) |
| 90 | 58.1 | 0.61 (0.25,0.98) | 0.92 (0.48,1.36) | 0.73 (0.45,1.01) | 0.71 (0.50,0.91) | 0.76 (0.58,0.94) |
| 75 | 53.1 | 0.94 (0.57,1.31) | 0.91 (0.74,1.08) | 0.90 (0.77,1.03) | 0.88 (0.77,1.00) | 0.91 (0.82,1.00) |
| 50 | 46.6 | 1 (reference) | 1 (reference) | 1 (reference) | 1 (reference) | 1 (reference) |
| 25 | 39.5 | 0.86 (0.48,1.24) | 1.21 (0.92,1.50) | 1.05 (0.85,1.25) | 1.04 (0.85,1.23) | 1.06 (0.88,1.23) |
| 10 | 33.1 | 0.98 (0.52,1.43) | 1.41 (0.97,1.84) | 1.21 (0.93,1.49) | 1.15 (0.91,1.39) | 1.22 (1.00,1.44) |
| 5 | 29.3 | 1.12 (0.50,1.74) | 1.53 (0.96,2.10) | 1.34 (0.95,1.72) | 1.24 (0.92,1.56) | 1.37 (1.06,1.67) |

*^1^* Values are hazard ratios (95%CI) for CVD at specific percentiles of the total HEFI-2019 score distribution, based on usual intakes. The hazard ratios are based on a fully adjusted Cox regression model using inverse probability weighting for exposure and death-censoring. The reference HEFI-2019 score (median) corresponds approximately to the HEFI-2019 participants had on average, i.e., under no hypothetical dietary change. The 95%CI were estimated using 250 bootstrap samples. HEFI-2019, Healthy Eating Food Index 2019.

## Risks based on a fully parametric modelling of time

An interaction test between the (log) time to CVD and the total HEFI-2019 score coefficient did not contradict the assumption of proportional hazards in the Cox regression model (p-interaction=0.69). However, such statistical test may be underpowered (18). Thus, the survival curves from a model using a fully parametric approach to model the time to CVD variable are presented below (**Supplementary Figure 3**). The corresponding risk estimates are presented in **Supplementary Table 13**.

**Supplementary Figure 3.** Probability of remaining CVD-free (survival curves) at varying HEFI-2019 score percentiles in adults from the UK Biobank. The probability of remaining CVD-free at the median HEFI-2019 score (yellow) is the mean probability and hence is the reference survival curve in a hypothetical scenario where there is no change in the HEFI-2019 in this population. Other survival curves reflect the probability of remaining CVD-free under hypothetical scenarios where all participants would achieve a HEFI-2019 score corresponding to predetermined percentiles in this population. Estimates of survival probability are based on fully adjusted pooled logistic regression models using inverse probability weighting for dietary exposure and death-censoring. In the pooled logistic regression model, the time to CVD was modelled using a restricted cubic spline with 5 knots and an interaction term between the total HEFI-2019 score and time to CVD was included. Total energy intake was also included in the pooled logistic regression model. The total HEFI-2019 score is based on usual dietary intakes modeled using the National Cancer Institute’s multivariate algorithm (see Methods). CVD, cardiovascular disease, HEFI-2019, Healthy Eating Food Index-2019.

**Supplementary Table 13.** Estimated risks of CVD in hypothetical scenarios where all eligible participants (n=132,777) in the UK Biobank achieve predetermined percentiles of the total HEFI-2019 score at baseline^1^

| **Total HEFI-2019***^2^* | | | **11-year CVD risk** | **Difference in risk estimates (95%CI)** | | |
| --- | --- | --- | --- | --- | --- | --- |
| Percentile | Score (/80) | Hypothetical change |  | Absolute, % point | Relative |  |
| 95 | 60.7 | + 14.1 pts | 1.7% | -0.75 (-1.47, -0.03) | 0.69 (0.42, 0.95) |  |
| 90 | 58.1 | + 11.5 pts | 1.8% | -0.59 (-1.09, -0.09) | 0.75 (0.57, 0.93) |  |
| 75 | 53.1 | + 6.5 pts | 2.1% | -0.26 (-0.49, -0.04) | 0.89 (0.80, 0.98) |  |
| 50 | 46.6 | 0 pts (reference) | 2.4% | 0 (reference) | 1 (reference) |  |
| 25 | 39.5 | - 7.1 pts | 2.7% | 0.26 (-0.23, 0.74) | 1.11 (0.93, 1.37) |  |
| 10 | 33.1 | - 13.5 pts | 3.1% | 0.66 (0.08, 1.25) | 1.28 (1.05, 1.63) |  |
| 5 | 29.3 | - 17.3 pts | 3.4% | 0.99 (0.25, 1.74) | 1.41 (1.13, 1.89) |  |

^1^ Difference in risk estimates reflect risks, had all participants in the sample achieved a prespecified HEFI-2019 score percentile, compared with the risk at the median HEFI-2019 score among all participants, the reference scenario where there is no change in HEFI-2019 (i.e., `0 pts`). Estimates are based on fully adjusted pooled logistic regression models using inverse probability weighting for dietary exposure and death-censoring. In the pooled logistic regression model, the time to CVD was modelled using a restricted cubic spline with 5 knots and an interaction term between the total HEFI-2019 score and time to CVD was included as well as total energy intake. The inverse probability weighting model covariates were sex, age, region, Townsend deprivation index, university degree, employment, familial history of cardiovascular disease, menopausal status (female only), hormone replacement use (female only), smoking habits, physical activity level, alcohol consumption habits, sedentary time, body mass index, dietary supplement use, medication use, and self-reported risk factor (high cholesterol and/or high blood pressure). The 95%CI were estimated using 250 bootstrap samples. CVD, cardiovascular disease; HEFI-2019, Healthy Eating Food Index-2019; IPW, inverse probability weighting; NCI, National Cancer Institute.

^2^ The HEFI-2019 score is based on usual dietary intakes modelled using the National Cancer Institute multivariate algorithm (see Methods).

## Relationship between the HEFI-2019 and CVD, by sex

The interaction between the (log) time to outcome and sex in a Cox regression model revealed evidence against the assumption that hazards were proportional (p<0.001). To model time-varying hazards, we also used a pooled logistic regression model. The time to CVD was modelled using a restricted cubic spline with 5 knots and an interaction term between the total HEFI-2019 score and time to CVD. Total energy intake was also included in the model. The sex-specific survival curves from the pooled logistic regression model are presented in **Supplementary Figure 4**.

**Supplementary Figure 4.** Probability of remaining CVD-free (survival curves) at varying HEFI-2019 score percentiles in males (A) and females (B) from the UK Biobank. The probability of remaining CVD-free at the median HEFI-2019 score (yellow) is the mean probability and hence is the reference survival curve in a hypothetical scenario where there is no change in the HEFI-2019 in this population. Other curves reflect the probability of remaining CVD-free under hypothetical scenarios where all participants would achieve a HEFI-2019 score corresponding to predetermined percentiles in this population. Estimates of survival probability are based on fully-adjusted pooled logistic regression models using inverse probability weighting for dietary exposure and death-censoring and stratified by sex. The inverse probability weighting models were also stratified by sex. In the pooled logistic regression model, the time to CVD was modelled using a restricted cubic spline with 5 knots and an interaction term between the total HEFI-2019 score and time to CVD was included. Total energy intake was also included in the pooled logistic regression model. The total HEFI-2019 score is based on usual dietary intakes modeled using the NCI multivariate algorithm (see Methods). CVD, cardiovascular disease; HEFI-2019, Healthy Eating Food Index-2019; NCI, National Cancer Institute.

## Potential effect of unmeasured confounding (E-Value)

While the no unmeasured confounding assumption can never be fully verified with observational data, the E-value can be useful to assess how large the contribution of an unmeasured confounder would have to be to explain the observed results (19). For the observed relative risk (RR) at the 90^th^ percentile of the HEFI-2019 score distribution compared with the reference (RR: 0.76; 95%CI: 0.58, 0.94), the E-value was 1.95 for the point estimate and 1.32 for the upper bound of the 95%CI. This implies that an unmeasured confounder would have to be associated with both the HEFI-2019 score and incident CVD by a risk ratio of 1.95-fold and 1.32-fold each to “nullify” the risk estimate and the upper 95% confidence bound, respectively. In other words, residual confounding would have to be relatively important to completely nullify the RR estimate of 0.76. However, relatively weak confounding could make the upper 95% bound (0.94) cross the null value of 1.00.

# Supplementary References

1. Young JG, Stensrud MJ, Tchetgen Tchetgen EJ, Hernan MA. A causal framework for classical statistical estimands in failure-time settings with competing events. Stat Med [Internet]. 2020;39(8):1199–236. Available from: <https://www.ncbi.nlm.nih.gov/pubmed/31985089>

2. Brassard D, Elvidge Munene LA, St Pierre S, Guenther PM, Kirkpatrick SI, Slater J, et al. Development of the Healthy Eating Food Index (HEFI)-2019 measuring adherence to Canada’s Food Guide 2019 recommendations on healthy food choices. Applied Physiology, Nutrition, and Metabolism [Internet]. 2022 Jan 14; Available from: <http://dx.doi.org/10.1139/apnm-2021-0415>

3. Brown IJ, Dyer AR, Chan Q, Cogswell ME, Ueshima H, Stamler J, et al. Estimating 24-hour urinary sodium excretion from casual urinary sodium concentrations in western populations: The INTERSALT study. Am J Epidemiol [Internet]. 2013;177(11):1180–92. Available from: <https://www.ncbi.nlm.nih.gov/pubmed/23673246>

4. Perez-Cornago A, Pollard Z, Young H, Uden M van, Andrews C, Piernas C, et al. Description of the updated nutrition calculation of the oxford WebQ questionnaire and comparison with the previous version among 207,144 participants in UK biobank. Eur J Nutr [Internet]. 2021;60(7):4019–30. Available from: <https://www.ncbi.nlm.nih.gov/pubmed/33956230>

5. Huang TT, Roberts SB, Howarth NC, McCrory MA. Effect of screening out implausible energy intake reports on relationships between diet and BMI. Obes Res [Internet]. 2005;13(7):1205–17. Available from: <https://www.ncbi.nlm.nih.gov/pubmed/16076990>

6. Black AE, Cole TJ. Within- and between-subject variation in energy expenditure measured by the doubly-labelled water technique: Implications for validating reported dietary energy intake. Eur J Clin Nutr [Internet]. 2000;54(5):386–94. Available from: <https://www.ncbi.nlm.nih.gov/pubmed/10822285>

7. Zhang S, Carroll RJ, Midthune D, Guenther PM, Krebs-Smith SM, Kipnis V, et al. A new multivariate measurement error model with zero-inflated dietary data, and its application to dietary assessment. The Annals of Applied Statistics [Internet]. 2011 Jun 1;5(2B). Available from: <http://dx.doi.org/10.1214/10-AOAS446>

8. Thompson FE, Kirkpatrick SI, Subar AF, Reedy J, Schap TE, Wilson MM, et al. The National Cancer Institute’s Dietary Assessment Primer: A Resource for Diet Research. Journal of the Academy of Nutrition and Dietetics [Internet]. 2015 Dec;115(12):1986–95. Available from: <http://dx.doi.org/10.1016/j.jand.2015.08.016>

9. Desquilbet L, Mariotti F. Dose‐response analyses using restricted cubic spline functions in public health research. Statistics in Medicine [Internet]. 2010;29(9):1037–57. Available from: <https://doi.org/10.1002/sim.3841>

10. Cole SR, Hernán MA. Adjusted survival curves with inverse probability weights. Computer Methods and Programs in Biomedicine [Internet]. 2004 Jul;75(1):45–9. Available from: <http://dx.doi.org/10.1016/j.cmpb.2003.10.004>

11. Hernán MA, Hernández-Díaz S, Robins JM. A Structural Approach to Selection Bias. Epidemiology [Internet]. 2004 Sep;15(5):615–25. Available from: <http://dx.doi.org/10.1097/01.ede.0000135174.63482.43>

12. Cole SR, Hernan MA. Constructing Inverse Probability Weights for Marginal Structural Models. American Journal of Epidemiology [Internet]. 2008 Jul 15;168(6):656–64. Available from: <http://dx.doi.org/10.1093/aje/kwn164>

13. Naimi AI, Moodie EE, Auger N, Kaufman JS. Constructing inverse probability weights for continuous exposures: A comparison of methods. Epidemiology [Internet]. 2014;25(2):292–9. Available from: <https://www.ncbi.nlm.nih.gov/pubmed/24487212>

14. Robins JM, Hernán MÁ, Brumback B. Marginal Structural Models and Causal Inference in Epidemiology. Epidemiology [Internet]. 2000 Sep;11(5):550–60. Available from: <http://dx.doi.org/10.1097/00001648-200009000-00011>

15. Kyle RP, Moodie EEM, Klein MB, Abrahamowicz M. Evaluating flexible modeling of continuous covariates in inverse-weighted estimators. Am J Epidemiol [Internet]. 2019;188(6):1181–91. Available from: <https://www.ncbi.nlm.nih.gov/pubmed/30649165>

16. Brakenhoff TB, Smeden M van, Visseren FLJ, Groenwold RHH. Random measurement error: Why worry? An example of cardiovascular risk factors. Sichieri R, editor. PLOS ONE [Internet]. 2018 Feb 9;13(2):e0192298. Available from: <http://dx.doi.org/10.1371/journal.pone.0192298>

17. Hernan MA. The hazards of hazard ratios. Epidemiology [Internet]. 2010;21(1):13–5. Available from: <https://www.ncbi.nlm.nih.gov/pubmed/20010207>

18. Stensrud MJ, Hernán MA. Why Test for Proportional Hazards? JAMA [Internet]. 2020 Apr 14;323(14):1401. Available from: <http://dx.doi.org/10.1001/jama.2020.1267>

19. VanderWeele TJ, Ding P. Sensitivity analysis in observational research: Introducing the e-value. Ann Intern Med [Internet]. 2017;167(4):268–74. Available from: <https://www.ncbi.nlm.nih.gov/pubmed/28693043>
